# Supplementary material for: Providing sex and relationships education for looked-after children: a qualitative exploration of how personal and institutional factors promote or limit the experience of role ambiguity, conflict and overload among caregivers
Source: BMJ Open. 2019 Apr 11;9(4):e025075. doi: 10.1136/bmjopen-2018-025075 (PMC6500194; doi:10.1136/bmjopen-2018-025075)
Supplement: Supplementary data [file bmjopen-2018-025075supp001.pdf]

Supplementary File 1: Interview Schedule

<u>Introductions</u>

Can you tell me a little bit about your experience working with/caring for looked after young people?

Explore: Type of contact with young people

Length of time working with them

Current Role

Reasons for working with the group

<u>Views of sexual health issues affecting young people in care</u>

Thinking about sexual health, can you tell me what you think are the most important issue affecting young people in care?

Explore: Pubertal changes, gender difference and menarche

Emotions and self esteem

Boyfriends and girlfriends

Choosing when to have sex and delaying sex

Contraception

STIs

Teenage pregnant/parenthood

Circumstances surrounding sex (e.g. risky, drugs/alcohol, force, consent)

Regret

Sexual health education

Service use

What/who do you think influences the sexual health of young people in care?

Explore: Type of care placement

Stability of placement

Security

Influence of peers/partners/parents/carers/corporate family

Media

Other

Sexual health education

Where do you think that looked after young people learn about sexual health and relationships?

Explore: School

Family

Friends/peers

Media

Social workers

Carers

Other

Thinking about the looked after young people and care leavers you know of look after, do you know what they know about sexual health and relationships?

Explore: What do they know?

What don't they know?

Where have they learned that information from?

What do you think about the quality of the information they received?

Have you spoken to them about sexual health and relationships?

- Why/Why not?

What have you spoken to them about?

Do you think there are barriers to young people in care accessing sexual health and relationships education?

Explore: What are those barriers?

What could be done to overcome the barriers?

Whose responsibility should that be?

Do you think that it is important for LAC to learn about sexual health and relationships?

Explore: Why/why not?

What do you think are the most important things for them to learn about? Why?

What would you change about the way young people in care learn about sexual health and relationships? Why do you think that?

Help, support and guidance around sexual health and relationships

What support should young people in care need in relation to their sexual health and relationships?

Explore: Pubertal changes and menarche

Boyfriends/girlfriends

Relationships

Choosing to have sex and sexual delay

Getting and using contraception

Using sexual health services

Pregnancy

Abortion

Other topics/advice/kinds of support not mentioned

Have you ever delivered support to a young person in care/care leaver in relation to their sexual health? Can you tell me about that in more detail?

How did delivering that support make you feel?

Did you feel that you had received sufficient training to be able to talk to a young person about their sexual health?

Explore: What training/support have you received?

What training/support would you have needed?

Who should have given you that support?

What effect do you think the training had/would have had upon your ability to support the young person you were speaking to?

What effect do you think you advice had upon the young person's…

Explore: Self confidence and self esteem

Knowledge

Relationships with boyfriends/girlfriends

Sexual relationships

Their relationship with you

Sexual health service use

What do you think are the barriers to young people in care accessing sexual health services?

If a young person came to you asking for information about sexual health services, could you give the that information? Where would you direct them to? Why? Would you accompany them there if they wanted you to?

What do you think a sexual health service for looked after young people should be like?

Explore: Staff

Accessibility

Opening hours/appointments

Location/entrance

Confidentiality

Other

Improving outcomes

What could be done to promote positive sexual health outcomes for looked after young people?

Why do you think that?
