# Supplementary material for: Providing sex and relationships education for looked-after children: a qualitative exploration of how personal and institutional factors promote or limit the experience of role ambiguity, conflict and overload among caregivers
Source: BMJ Open. 2019 Apr 11;9(4):e025075. doi: 10.1136/bmjopen-2018-025075 (PMC6500194; doi:10.1136/bmjopen-2018-025075)
Supplement: Author's manuscript [file bmjopen-2018-025075.draft_revisions.pdf]

BMJ Open is committed to open peer review. As part of this commitment we make the peer review history of every article we publish publicly available.

When an article is published we post the peer reviewers' comments and the authors' responses online. We also post the versions of the paper that were used during peer review. These are the versions that the peer review comments apply to.

The versions of the paper that follow are the versions that were submitted during the peer review process. They are not the versions of record or the final published versions. They should not be cited or distributed as the published version of this manuscript.

BMJ Open is an open access journal and the full, final, typeset and author-corrected version of record of the manuscript is available on our site with no access controls, subscription charges or pay-per-view fees (http://bmjopen.bmj.com).

If you have any questions on BMJ Open's open peer review process please email info.bmjopen@bmj.com

BMJ Open

Providing sex and relationships education for looked after children: a qualitative exploration of how personal and institutional factors promote or limit the experience of role ambiguity conflict and overload among caregivers

Journal:	*BMJ Open*
Manuscript ID	bmjopen-2018-025075
Article Type:	Research
Date Submitted by the Author:	28-Jun-2018
Complete List of Authors:	Nixon, Catherine; University of Glasgow, MRC/CSO Social and Public Health Sciences Unit<br>Elliott, Lawrie; Glasgow Caledonian University, Department of Nursing and Community Health<br>Henderson, Marion ; University of Glasgow, MRC/CSO Social and Public Health Sciences Unit
Keywords:	looked-after children, sexual health, caregivers, social workers, QUALITATIVE RESEARCH

SCHOLARONE™
Manuscripts

For peer review only

Providing sex and relationships education for looked after children: a qualitative exploration of how personal and institutional factors promote or limit the experience of role ambiguity conflict and overload among caregivers

For peer review only

Catherine Nixon[1*], Lawrie Elliott[2] and Marion Henderson[1]

Correspondence to:

Dr. Catherine Nixon

MRC/CSO Social and Public Health Sciences Unit, University of Glasgow, 200 Renfield Street, Glasgow, G2 3QB, Scotland, UK

catherine.nixon@glasgow.ac.uk

Keywords: looked-after children, sexual health, caregivers, social workers, qualitative research

Word count: 5986 (4750 excluding participant quotes)

For peer review only - http://bmjopen.bmj.com/site/about/guidelines.xhtml

Abstract

Objectives: To explore how personal and institutional factors promote or limit the role of caregivers in promoting sexual health and relationships (SHR) among looked-after children (LAC).

Design: Qualitative semi-structured interview study.

Setting: UK social services, residential children's homes and foster care.

Participants: 22 caregivers of LAC, including 9 foster carers, 8 residential carers and 5 social workers; half of whom had received SHR training.

Methods: In-depth interviews explored barriers/facilitators to SHR discussions, and how these shaped caregivers' experiences of discussing SHR. Data were systematically analysed using predetermined research questions and themes identified from reading transcripts. Role theory was used to explore caregivers' experiences of their role.

Results: SHR education policies clarified role expectations and increased acceptability of SHR discussions. Training increased knowledge and confidence, and supported caregivers to reflect on how personally held values impacted their practice. Identified training gaps were how to: 1) discuss SHR with LAC demonstrating problematic sexual behaviours and 2) record that SHR discussions had occurred in LAC's health plans. Contrary to previous findings, caregivers regularly discussed SHR with LAC. Competing demands upon time resulted in prioritisation of discussions for sexually active LAC and those 'at risk' of sexual exploitation/harm. Interagency working was used to address gaps in SHR education provision. SHR discussions placed emotional burdens upon caregivers. Caregivers worried about allegations being made against them by LAC. Managerial/pastoral support and 'safe care' procedures were used to minimise these harms.

Conclusions: Whilst acknowledging the existing level of sexual health promotion for looked-after young people there is scope to have this more firmly embedded into the role of caregivers.. Care needs to be taken to avoid role ambiguity and tension when

For peer review only

For peer review only - http://bmjopen.bmj.com/site/about/guidelines.xhtml

doing so. Providing SHR policies and training, and promoting interagency working and the use of pastoral support are important steps towards achieving this.

Article summary

Strengths and limitations of this study

- Participant accounts provided rich qualitative data that provided insight into how the introduction of SHR training and policies affected the undertaking of sexual health and relationships discussions with looked-after children.
- Role theory was used to interpret how the introduction of locally provided policies and trainings promoted or limited caregivers' experiences of discussing sexual health and relationships.
- Our study does not capture the experiences of kinship carers or parents whose children may be looked-after in their family home under the terms of a home supervision order.
- The findings of this study may not be generalisable to the practices of caregivers outwith Scotland due to the small non-random sample and differences in the political and legal frameworks underscoring the care and protection of children.

For peer review only

For peer review only - http://bmjopen.bmj.com/site/about/guidelines.xhtml

Introduction

Children who are 'looked-after' are an important group for public health intervention. In the UK a child is considered to be a 'looked-after child' (LAC) if parental responsibility is assumed by social services or shared between the child's parents and social services. LAC can experience a variety of care arrangements, including: foster care; placement in residential (children's) homes, residential schools or secure units; being looked-after by relatives or family friends in kinship placements; and being looked-after at home with their parents under the supervision of social services [1, 2]. In 2016-2017, approximately 100,000 children were looked-after by local authorities in the four UK nations [3-6]. Whilst children may enter care as a result of their own or parental illness/disability, the majority become looked-after as a result of neglect or abuse [6, 7].

Childhood maltreatment and pre-care experiences characterised by poverty, family breakdown, domestic violence, and parental mental illness and/or substance abuse increase LAC's vulnerability to experiencing: mental health difficulties; school exclusion; low educational attainment; substance use; and, antisocial/offending behaviour [8-11]. Cumulative exposure to these factors, which are independently associated with increased sexual risk-taking [12-16], mean LAC experience poorer sexual health outcomes than adolescents who have never been looked-after. For example, studies from the UK, USA and Sweden have demonstrated increased rates of early sexual activity, greater numbers of lifetime sexual partners, poorer contraceptive use, and increased rates of sexually transmitted infections (STIs), teenage pregnancy/parenthood, sexual exploitation/assault and rape [17-23].

For peer review only - http://bmjopen.bmj.com/site/about/guidelines.xhtml

For peer review only

The poor sexual health of LAC may be exacerbated by limited access to sexual health and relationships (SHR) education. Barriers to SHR education include increased school absence/exclusion rates, frequent placement moves and poor-quality relationships with parents [24-26]. SHR discussions in care settings are also limited [24, 27-29]. For example, a survey of Californian social workers (SWs) reported that 66–77% had never spoken to LAC about puberty, the characteristics of healthy relationships, abstinence, contraception, sexual orientation, STIs, pregnancy/parenthood and abortion. [28]. Reported barriers to caregivers discussing SHR include: lack of SHR knowledge and training; difficulties reconciling personal religious/moral values; lack of guidance about who is responsible for discussing SHR; uncertainty as to whether SHR discussions infringe upon the rights of biological parents; concerns that SHR discussions condone sexual activity; uncertainty about the boundaries between confidentiality and child protection (CP); and concerns about LAC making allegations of wrongdoing against caregivers [27, 28, 30, 31].

Calls have been made to improve LAC's access to SHR education [32-34]. Existing evidence shows that adolescents whose parents discuss SHR with them are more likely to delay intercourse, use contraception and have fewer sexual partners [35-38], and it has been recommended that caregivers discuss SHR with LAC. In Scotland, these recommendations have resulted in social work departments developing SHR policies and working with health-boards to provide SHR training to caregivers [39, 40].

Role theory [41-43] states that practitioners develop specific beliefs and attitudes about their profession which establish the boundaries of their role and how they interact with other professionals, and those they serve [44]. Introducing new responsibilities can challenge these beliefs and blur the boundaries of the existing roles. [45]. While role blurring has advantages, such as allowing practitioners to adopt new responsibilities and be more responsive to the needs of the population they are working with, it can result in them feeling overwhelmed and uncertain about the limits of their new role [46]. Role

For peer review only - http://bmjopen.bmj.com/site/about/guidelines.xhtml

blurring can also introduce sources of conflict, additional workload and ambiguity to their professional role [44, 47].

Role conflict occurs when practitioners attempt to fulfil two or more roles with incompatible expectations, requirements, beliefs or attitudes. For example, healthcare practitioners with strong personal views about the acceptability of abortion may find it difficult to provide information or advice about terminating an unwanted pregnancy [48, 49]. Role overload occurs when the demands of a particular role exceed the capacity of the individual [44, 47]. Finally, role ambiguity occurs when there is a lack of guidance/clarity about roles, and can result in: practitioners being unclear about what is expected of them; create concerns about duplication of work; and, act as a source of frustration, tension or anxiety at work [47].

In this study we explore how caregivers' of looked-after young people construe their role as promoters of sexual health. To do this, we used role theory [41-43] as a framework to answer the following research question: 'how do personal and institutional factors promote or limit the experience of role ambiguity, conflict and overload among caregivers tasked with discussing SHR with LAC?' In addressing this question we extend the existing evidence base by moving beyond identifying barriers to SHR discussions being undertaken within the care system [24, 27-29] and providing new insights into how SHR discussions can be incorporated into practice.

Methods

In-depth individual qualitative interviews were conducted with 22 caregivers from August-October 2011. As we wanted to explore how SHR discussions were influenced by caregivers' perceptions of their role, our sample included three groups of caregivers: foster carers (FC; n=9), residential carers (RC; n=8) and SWs (n=5). All caregivers were recruited from a large urbanised local authority in Scotland, which had recently

For peer review only - http://bmjopen.bmj.com/site/about/guidelines.xhtml

introduced SHR training for caregivers. Respondents were randomly selected from staff lists stratified by whether caregivers had received SHR training or not. Our aim was to interview caregivers when half of all FCs, RCs and SWs had received training; however, institutional delays in rolling out training meant that interviews were undertaken when half the FCs, all RCs and no SWs had received training. Our achieved sample reflects this. Interviews focussed on: SHR training; experiences of providing advice about SHR; perceived barriers/facilitators to undertaking SHR; which SHR topics were discussed; and how discussions were shaped by the experiences of LAC and their caregivers.

Interviews were digitally recorded, transcribed verbatim, anonymised and entered into NVivo 9 (QSR International, Melbourne, Vic, Australia) for data management and coding. Data were analysed thematically. A coding framework was developed based on both predetermined research questions and themes identified through close reading of interview transcripts. A selection of transcripts and the coding framework were then reviewed by independent reviewers, and the coding framework applied to all transcripts once finalised. This allowed systematic comparisons to be made across the data and facilitated the identification of recurrent themes. Emergent themes were categorised by whether they acted as barriers or facilitators to the inclusion of SHR discussions within the professional role. Using role theory [41-43] as a framework for interpreting our findings, we also categorised emergent themes to show how introducing SHR discussions into practice contributed to experiences of role ambiguity, conflict and overload. Reflections upon how researcher characteristics may have influenced the research are reported elsewhere [50]. SRQR reporting guidelines [51] were used to oversee the design, analysis and reporting of findings; details per supplementary file 1.

Ethical approval

Ethical approval was granted by Glasgow University's Faculty of Law, Business and Social Sciences Ethics Board [Application number: SS/10/0027]. The research tools were also

For peer review only - http://bmjopen.bmj.com/site/about/guidelines.xhtml

reviewed and approved by the Director of Children and Family Services on behalf of the local authority.

Patient involvement

Patients were not involved in the design of this study.

Results

Our results highlight that inclusion of SHR interactions within caregiving is influenced by the interplay of institutional factors and caregivers' personal values/experiences. Table 1 provides a thematic overview of these factors and how they contributed to the experience of role ambiguity, conflict and overload.

Role ambiguity

SHR policies reduced role ambiguity and increased the acceptability of SHR discussions among RC and SW by addressing historical taboos about discussing sex and emphasising the inclusion of SHR discussions within caregivers' professional role: *"it's a priority now... it's something that's been brought to the forefront, where it's no considered taboo" (Shona, SW/relief RC).* In particular, SHR policies emphasised that undertaking SHR discussions allowed caregivers to fulfil their "corporate parenting" responsibilities. Being perceived to be a 'good' corporate parent emphasised RCs and SWs perceptions of their professional role, and hence, SHR interactions were identified as being part of routine caregiving: *"you would talk to your kids about it (sex). And that's what we do as corporate parents. We take on that role and responsibility" (Rachel, SW).* These effects were not reported by FCs: *"we don't shy away. If there was a sex scene in a movie or whatever, we quite often discuss it rather than say 'oh my goodness, we shouldn't be watching that, hide your eyes boys!' We just relax about it. It's not something that we try to pretend isn't there" (Alison, FC).*

For peer review only - http://bmjopen.bmj.com/site/about/guidelines.xhtml

For peer review only

SHR policies clarified what was expected of caregivers when discussing SHR with LAC. This clarification was welcomed by several RCs who had been reprimanded *("I got my bum booted, Joanne RC)* for doing things such as accompanying LAC to obtain emergency contraception. Whilst on the whole SHR policies reduced role ambiguity, lack of guidance on specific elements also contributed to role ambiguity. One example of this was how SHR discussions should be recorded, with RCs perceiving that they did not have clear guidance about how to confidentially record SHR discussions when there were no Child Protection concerns. Lack of confidentiality was perceived as a barrier to LAC approaching caregivers for help: *"recording it is very difficult. We have general comms [communication] books which are for everybody's viewing which is not appropriate, and we have individual logs which aren't appropriate either because the kid is maybe keen to keep something in confidence but then it is written down. It is a grey area" (Mark, RC).*

Role conflict

Including SHR interactions into caregiving practices resulted in two sources of role conflict that acted as barriers to SHR. These were: the challenges of balancing the competing responsibilities of managing child protection concerns with preventative SHR; and reconciling professional responsibilities for discussing SHR with personally held religious/moral views about sexual behaviours. Factors that reduced role conflict and facilitated SHR interactions included: developing strategies to monitor LAC's sexual behaviours and protect against the risk of allegations being made; caregivers having the experiences of discussing SHR with their biological children to help frame discussions with LAC; and caregivers reflecting upon the school- and home-based SHR education that they had received as motivation for undertaking SHR discussions. These themes were predominantly raised by RCs and FCs.

Risk management and SHR discussions

RCs and FCs experienced challenges balancing the competing demands of CP concerns with undertaking preventative SHR interactions. Many of those interviewed stated that

For peer review only - http://bmjopen.bmj.com/site/about/guidelines.xhtml

their role often focussed on policing/supervising sexual behaviour than providing SHR information: *"I was no longer a caregiver – I was a security guard" (Karen, FC/former RC).* As such, SHR discussions were often undertaken in response to specific safety concerns: *"there wouldn't always be a major, in-depth discussion if there weren't any major issues… but if a child is sexually active, and they're underage, and… running away, having sex wi' [with] men they don't know, coming back the next morning covered in mud, drinking… it would be very high on the agenda" (Agnes, SW).* Managing risk was particularly emphasised by those caring for LAC who had experienced sexual exploitation/abuse and/or were demonstrating sexually inappropriate or sexually abusive behaviours towards others. Caregivers identified a range of strategies they used to manage the sexual health of LAC, including: monitoring friendships; monitoring mobile phone and computer usage; assessing the risks associated with young people in their care going on unsupervised outings; and carefully managing internal public spaces and room allocations. For example, Joanne (RC) described how they had recently reallocated bedrooms so that a girl in placement who "*would encourage the rest of the group to go out drinking, and then make allegations of rape against one or more of the boys*" was located closest to the office so she could receive additional monitoring. Monitoring risk, particularly for LAC demonstrating inappropriate sexual behaviours, understandably affected caregivers' emotional wellbeing. For example, one FC (Jean) became upset and started visibly shaking while recounting an incident where she had found two siblings she cared for *"in the sliding wardrobe, a pillow put down on the inside to mask the noise... and the girl on the floor as he was on top of her holding her down".*

Monitoring the sexual health of LAC acted as a source of conflict when caregivers had to balance the SHR needs of LAC against protecting the safety, wellbeing and livelihoods of other household and family members. For example, several FCs discussed how allegations of improper behaviour could have financial/reputational repercussions, and affect the other roles they fulfilled: *"it's worrying… my son's a police officer, my husband works in law enforcement and I work with students –we have to take extra care" (Alison,*

For peer review only - http://bmjopen.bmj.com/site/about/guidelines.xhtml

FC). Caregivers' concerns affected how SHR discussions were undertaken, with all of those interviewed emphasising the importance of using 'safe care' practices to minimise the risk of harm to caregivers. These practices included having conversations in private but open spaces, having another caregiver present and ensuring that discussions were accurately documented. Safe care practices were also used to minimise the risks of harm to other individuals living/working within placement, and shaped the SHR topics routinely discussed with LAC. These topics included privacy, body autonomy, appropriate clothing, modesty and developing/enforcing rules around bedroom and bathroom privacy: *"you need to keep reinforcing what is and is not appropriate behaviour… it is not appropriate to be showing yourself off. It's not appropriate to be going into the toilet with other boys" (Karen, FC).*

Drawing upon and reconciling personal experiences

Some caregivers experienced role conflict when they attempted to reconcile personally held religious/moral beliefs about sex with the professional responsibility of talking to LAC about sexual feelings, masturbation, contraception and sex: *"I'm a practising catholic. It has took me an awful long time tae do all my challenging in myself and asking and prying aboot [about] how does that fit with my psyche to sit here and talk about things that I ordinarily would not talk aboot" (Claire, RC).* Being provided with time in SHR training sessions to reflect upon how personal values could affect SHR discussions was identified as a way of addressing this conflict. Pastoral support was also identified as a means of supporting caregivers experiencing difficulties reconciling personal views with professional expectations. For example, one RC described supporting a fellow caregiver who strongly opposed abortion to "*present all the options*" to a "*girl in her care who wanted to terminate the pregnancy*".

While personal experiences acted as a source of conflict for some caregivers, for others, personal experiences reduced role conflict and facilitated SHR interactions. For example, caregivers' own experiences of not receiving school- or home-based SHR education as a

For peer review only - http://bmjopen.bmj.com/site/about/guidelines.xhtml

child reduced conflict by providing motivation for SHR discussions being incorporated into practice: *"I went to college at sixteen and… I'm sitting in a class and I'm looking at this film on childbirth and I see where a baby's born from. I thought that they untied your tummy button, took it out, tied it up again and stuck it back in. Now I did bring my children up... from when they were wee tots… I would get them to go and get my sanitary towels and I would tell them what it was" (Patricia, FC).* Having discussed SHR with a biological child reduced conflict by providing caregivers with experiences to draw upon when talking to LAC: *"it was always just a natural kind of growing up. We spoke about contraception, and my daughter, I was able to go with her to the doctors when she wanted to start taking the pill. We could just talk about it really openly. Likewise, with Michael [foster child], we've approached the subject of puberty and changes in the body" (Alison, FC).*

Workload

Not having sufficient workforce capacity acted as a barrier to SHR interactions. Lack of capacity arose in three ways: not having sufficient numbers of caregivers to allow for SHR interactions to be undertaken; not having the time to build up trusting relationships with LAC; and not having the necessary skills/knowledge to undertake SHR discussions. Factors that reduced workload and facilitated SHR interactions included: interagency and partnership working; SHR training; and receiving pastoral support from management and colleagues.

Workforce capacity, interagency working and building relationships

Having insufficient numbers of caregivers reduced RCs capacity to undertake SHR education. Collaborating with external agencies was identified as reducing role overload and ensuring that LAC received help and support: *"we're limited wi' [with] staff... so it's a bit aboot [about] sharing you know? We kind of all come together" (Patricia, RC).* Conversely, partnership working introduced the potential to duplicate work. To avoid this, and to ensure LAC did not receive contradictory SHR messages, caregivers

For peer review only - http://bmjopen.bmj.com/site/about/guidelines.xhtml

highlighted the importance of being informed about the content of SHR education that was delivered by partner organisations: *"we've had reports from them and we know what they are doing… so we tend to back off and let one person do that work on sexual health and keeping safe" (Joanne, RC).*

The importance of interagency working was discussed by SWs, who reported that excessive workload acted as a barrier to their having sufficient time to discuss SHR with LAC. Time demands meant that SWs were more involved with the coordination rather than the provision of SHR education: *"a safety plan gets planned and implemented… and focused work carried out that is specific and tailored to that young person's needs and risks… that's something that as the allocated worker I would review and monitor".* Competing demands meant SWs did not have time to build up the trusting relationships LAC formed with RCs, FCs and keyworkers provided by external agencies: *"as a social worker it's a lot more difficult to really get to know that young person because in residential… you really get to know the young people because you see them for 24-hour periods, and you know a lot more about their life, and what's happening on a daily basis… being a social worker… there's a lot more hidden" (Lindsay, SW).* Lack of time, combined with perceptions that LAC and their families distrusted SWs, resulted in SWs reporting that RCs and FCs were better placed to discuss SHR with LAC: *"the best person to guide that young person in terms of their sexual development, their sexual health, is the person that they trust. And that's their carer" (Mike, SW).*

LAC were perceived to be more comfortable and less embarrassed discussing SHR with caregivers of the same sex: *"Matthew talks more about these things to my husband... after he has these chats you just see a different wee boy. You see him all relaxed". (Alison, FC).* The low proportion of male RCs was identified as both a potential barrier to male LAC receiving SHR information and a source of role overload for female RCs due to their being predominantly responsible for undertaking SHR discussions. Female caregivers perceived that male caregivers, as a result of their lived experiences, were

For peer review only - http://bmjopen.bmj.com/site/about/guidelines.xhtml

often better placed to explain or demonstrate sex-specific hygiene practices: *"teaching them how to shave for example, that's not something I can do. So, if I have a male worker, then I get him to come into work unshaven so he can show the boys how to shave properly" (Trisha, RC).*

SHR training

Not having the skills and training needed to undertake SHR discussions acted as a barrier to caregivers interactions on SHR with LAC. SHR training, however, was identified as a way to address this: *"if I was in the position of working with a young person who had a very trusting relationship with me, and who required support with their sexual health and development, then I would like to play a part in that… but I'd like training because I see that as a gap" (Mike, SW).* SHR training was reported to improve caregivers' knowledge of sexual health terminology, contraceptive choices, STIs and colloquialisms used by LAC to describe sexual acts: *"the course opens your eyes to it, you know? You can go through life thinking, well, right, ok, I know about Gonorrhoea and this kind of stuff, but I don't know about Chlamydia, and I don't know about this, that and the next thing. And these are all things that children can get, and I need to be able to explain what can happen if they have unprotected sexual relationships" (John, FC).* Being able to draw upon SHR knowledge improved caregivers' confidence and reduced embarrassment. Caregivers also emphasised how being able to use the correct terms reduced the possibility of discussions being misconstrued by LAC: *"one worker talked about your flower, and if you needed anything sorted you would go to the flower shop… I don't think that things like that really help when talking about going to clinics and your vulva… she could have people thinking 'oh right, I need to go buy some flowers'… because they take you literally" (Laura, RC).*

Caregivers who had received SHR training highlighted the importance of being provided with leaflets and books that they could use to help discuss SHR with LAC: *"after the training I found that I was really more confident and I had all the information on hand*

For peer review only - http://bmjopen.bmj.com/site/about/guidelines.xhtml

and booklets to show" (Maria, FC). They also discussed how the training had provided them with details of websites and helplines that they could use if they were asked questions that they did not know the answer to. Having these resources was valued as it allowed caregivers to talk about sex *"from a knowledge base" (Laura, RC).* Training resources helped caregivers tailor SHR discussions to the physical, emotional and cognitive ages of LAC: *"we did a great book that illustrated the changes in their body because obviously when they go in and they have baths and they have showers they're noticing their body changing and they become more inquisitive" (Alison, RC).* This was viewed as important, as often LAC who had experienced maltreatment did not have emotional and cognitive ages that reflected their physical age.

While SHR training provided caregivers with the skills needed to incorporate SHR education into practice, it should be noted that all RCs and FCs, regardless of whether they had received SHR training or not, routinely discussed SHR with LAC. The range of topics reported as being discussed within caregivers' narratives included puberty, peer relationships, romantic relationships, masturbation, having sex for the first time, contraception, reproduction and sexual exploitation. The main difference observed was that caregivers who had not received SHR training were more likely to express concerns about the accuracy of the information they had provided: *"we were playing Connect 4, and one of the girls said 'how do you get pregnant' and we said 'well, you need to have sex'. 'Aye, I know that… and I know that he cums, but how does that then work?' So we dismantled the Connect 4, and we said 'well it's no square, but you'll have to imagine this is a womb, and these are the fallopian tubes', and we used the wee circles as the sperms and the eggs, and we used that to explain it…. Once we were finished I turned to [another caregiver] and said 'did I get that right?'" (Claire, RC).*

Pastoral support

Pastoral support from managers and other caregivers helped caregivers engage with SHR education including those who felt unable to do so because of insufficient knowledge or training. Several caregivers stated that hearing about others' experiences of

For peer review only - http://bmjopen.bmj.com/site/about/guidelines.xhtml

discussing SHR with LAC in one-to-one or group supervision sessions allowed them to incorporate others' experiences into their practice: *"draw upon their experiences, information… and real examples" (Fiona, SW)*. Pastoral support was particularly valued when caregivers had to discuss SHR with LAC who had been sexually abused, groomed or exploited and were demonstrating problematic sexual behaviours: *"I found that work really difficult, because I had never had to deal with… trying to manage a child – cos he was a child at the time — who is not only, you know, being abused, but is an abuser… I felt really, you know, unsure of how best to manage that. One of the best things with managing that was that my manager agreed to support me, and we did the work together" (Fiona, SW).* The main reason for this was that caregivers found these discussions to be challenging and did not have the skills and training needed to undertake these discussions alone.

Discussion

Using role theory [41-43] we identified that the introduction of SHR education into LC settings contributes to role ambiguity conflict and in some instance excessive demands on caregivers. In doing so, we have extended existing knowledge by moving beyond characterising the reasons why SHR discussions within the care system are limited [24, 27-29] by providing insights into how SHR policy, training, interagency working and pastoral support can be used to foster SHR interactions between caregivers and the young people in their care. Our results show that lack of SHR training and knowledge, competing demands upon caregivers' time and limited workforce capacity contributed to the experience of role overload. Difficulties reconciling personal and professional views of SHR and balancing the competing responsibilities of child protection concerns and preventative SHR work contributed to the experience of role conflict, and placed emotional burdens upon caregivers. Lack of clear guidance about how to confidentially record SHR discussions contributed to the experience of role ambiguity. Together, these factors acted as barriers to SHR interactions occurring within LAC settings.

For peer review only - http://bmjopen.bmj.com/site/about/guidelines.xhtml

Our findings highlight that SHR policies and training remove barriers to SHR discussions occurring by; creating a working culture supportive of SHR discussions being incorporated into practice; providing caregivers with knowledge and confidence to talk about SHR; and supporting caregivers to reflect upon how their own personal experiences, morals and values impacted upon their practice. SHR discussions were further facilitated by the promotion of collaborative working, the use of safe care procedures and the availability of managerial and pastoral support.

Institutional support for intervention delivery contributes to the successful adoption of general health prevention programmes [52]. Our results demonstrate that this also applies to SHR education in LAC settings by reducing taboos about sex and creating a culture supportive of caregivers. In this study, SHR policies promoted culture change by emphasising that SHR discussions were part of caregivers' corporate parenting responsibility to support LAC to obtain the "same health outcomes… as any good parent would want for their own children" [53]. Being perceived to be a "good" corporate parent contributed strongly to the professional role identity of the caregivers we interviewed, which in turn increased acceptability of SHR discussions being incorporated into practice. This finding suggests that framing SHR policies to reflect the professional identity of caregivers can act as an important first step towards the successful incorporation of SHR discussions into practice.

Lack of guidance about who should be responsible for discussing SHR acts a barrier to LAC receiving SHR information and advice in non-LAC settings [27, 28, 30, 31]. Our results show that SHR policies in LAC settings can reduce role ambiguity by clarifying role expectations. This is important as previous studies in non-LAC settings have demonstrated that when role ambiguity occurs, both parents and health professionals choose not to discuss SHR with adolescents due to perceptions that there are others who are better qualified to undertake this role [54]. Lack of guidance on how to confidentially

For peer review only - http://bmjopen.bmj.com/site/about/guidelines.xhtml

record SHR discussions contributed to the experience of role ambiguity among caregivers. It also acted as a potential barrier to caregivers discussing SHR with LAC due to concerns that information sharing impacted upon LAC's rights to confidentiality. Lack of confidentiality is a known barrier to adolescents approaching services for SHR information [55], and therefore may prohibit LAC from engaging in open SHR discussions with caregivers. To minimise the risk of this happening, SHR policies and training should provide clear guidance on how and where caregivers should record SHR discussions, and outline the circumstances under which information sharing is required, namely where CP concerns exist [56]. This is particularly important for residential settings, where multiple caregivers require access to LAC's health information.

Previous studies have indicated that SHR discussions within LAC settings are limited [24, 27-29]; however, our results demonstrate that caregivers regularly discussed SHR with LAC. Nevertheless, some caregivers experienced role conflict as they attempted to reconcile their professional duty to discuss SHR with personally held religious/moral beliefs about sex. This resulted in some caregivers opting out of providing information on sensitive issues such as abortion; a finding echoed in other studies looking at healthcare practitioners' attitudes towards contraception and carrying out medical terminations of pregnancy [48, 49]. Some personal experiences, including caregivers having the experience of discussing SHR with their own biological children, reduced role conflict by providing caregivers with the confidence to undertake SHR discussions.

While having personal experiences to draw upon may facilitate SHR discussions, evidence from secondary schools shows that in the absence of SHR guidance and training, teachers incorporate their own moralistic views of sexual behaviour into SHR discussions with pupils [57, 58]. As it is known that adolescents do not seek SHR information from adults they perceive to be judgemental [59] it is important that caregivers provide LAC with factual, evidence-based information about SHR. To facilitate this, SHR training should provide caregivers with opportunities to reflect upon how their

For peer review only - http://bmjopen.bmj.com/site/about/guidelines.xhtml

personal views may affect how they undertake SHR discussions in practice. SHR policies and training should also identify support mechanisms for caregivers who experience difficulties reconciling their personal views about SHR into professional practice. This support could include the provision of managerial/pastoral support for undertaking SHR discussions or the opportunity for caregivers to reflect upon their experiences of role conflict in either group or 1-2-1 supervision sessions.

SHR policies and training should take into account the burdens that caring for LAC can place upon caregivers. Our results highlight that the demands that managing CP concerns place upon caregivers time contributes to role overload. This can result in preventative SHR discussions not being undertaken unless support from external agencies is sought or the role of meeting the sexual health needs of LAC is undertaken by specialist public health (LAC) nurses [30]. To avoid role ambiguity occurring, it is important that clear guidance is provided about the role that caregivers should play in the provision of SHR to LAC when external agencies are used to support LAC receiving SHR information and advice. To that end, the health plans of LAC should clearly outline: their sexual health needs; what information/intervention is required; and which individuals/agencies will be involved in meeting those needs. A lead professional should be identified and tasked with the responsibility of overseeing any multi-agency involvement, and ensuring that all individuals/agencies involved have clearly delineated roles and responsibilities [30].

Caring for LAC who have experienced sexual abuse/exploitation can result in vicarious trauma and strain [60, 61]. This was evident in our results. Identifying ways to minimise caregiving strain is important as it is known that caregiving strain can contribute to placement breakdowns [60, 62]. Repeated breakdowns of placements erode the ability of LAC to form secure and trusting relationships with their caregivers [63], which in turn may lead to LAC being unable to identify a consistent and trusted adult that they can discuss SHR with [25, 28, 64]. Caregiving strain can also negatively impact upon the

For peer review only - http://bmjopen.bmj.com/site/about/guidelines.xhtml

sensitivity of caregivers' parenting skills, particularly the ability to respond to the emotional rather than physical ages of children [60]. As the majority of LAC have experienced child maltreatment [6] and there are known associations between maltreatment, emotional dysregulation and cognitive delay [65], sensitive and attuned parenting skills are required to effectively tailor preventative SHR messages to the emotional and cognitive ages of LAC. One potential way of alleviating caregiving strain is through peer-led support services. For instance, recent evidence shows that the provision of peer support for parents with children experiencing mental health difficulties reduces isolation, increases social support, empowers caregivers and increases levels of self-care [66]. Future research should explore whether peer-led sexual health support services for caregivers can empower caregivers to undertake SHR discussions, while reducing caregiving strain.

Finally, our results emphasise the importance of SHR training for facilitating SHR discussions within the care setting. SHR training reduced role overload by providing caregivers with the knowledge needed to undertake SHR discussions with LAC. This in turn improved caregivers' confidence. Our findings reflect results from other studies of sexual health, which have demonstrated that the introduction of SHR training improves the confidence of teachers and medical professionals to talk with pupils and patients about SHR [67-69] and increases the likelihood of parents engaging in conversations about sex with adolescents [70]. It is encouraging that training facilitated SHR discussions, as evidence shows that adolescents whose parents discuss SHR with them are more likely to delay intercourse, use contraception and have fewer sexual partners [35-38]. Further research should establish whether caregiver-led SHR discussions can contribute to a reduction in sexual risk among LAC.

Strengths and limitations

Strengths of this qualitative interview study include the generation of rich qualitative data that provided insight into how the introduction of SHR training and policies

For peer review only - http://bmjopen.bmj.com/site/about/guidelines.xhtml

facilitated SHR discussions within the care setting, and how caregivers negotiated and addressed perceived impacts of undertaking SHR discussions upon their professional and personal identities. As LAC are often excluded from school-based SHR education, the data generated from this study provides valuable insights into alternative means through which SHR discussions can be undertaken within this population.

This study has four main limitations. First, this was an exploratory study with a relatively small sample of caregivers recruited from a single local authority, therefore generalising the findings from this study to the practices of other caregivers may not be possible. This may be particularly true when it comes to translating these findings into care settings in other countries due to differences in the political and legal frameworks underscoring the care and protection of children. Second, while our aim was to interview caregivers when half of all FCs, RCs and SWs had received SHR training, institutional delays in rolling out training to caregivers meant that interviews occurred when half the FCs, all RCs and no SWs had received training. While our achieved sample reflects this, it is possible that our findings for SWs in particular do not fully capture how being asked to discuss SHR with LAC challenges professional role identity. Third, our study does not capture the experiences of kinship carers or parents whose children may be looked-after at home under home supervision orders. Future research should explore how the experiences of these caregivers differs from that of other caregivers. The final limitation is that while we have used role theory as a theoretical framework for interpreting our findings, this study was not designed to evaluate the impact that introducing SHR discussions into practice had upon caregivers' constructions of their professional identity. To that end, it would be beneficial if future revisions of sexual health training and policy within the care system reflected evaluative work being undertaken in the field of nursing [71] and collected pre- and post- quantitative and qualitative data on the effect that introducing new policy has upon the constructions of caregivers' professional identity.

For peer review only

For peer review only - http://bmjopen.bmj.com/site/about/guidelines.xhtml

Conclusion

To encourage SHR discussions within the care setting it is important that organisations adopt a culture that is supportive of LAC receiving SHR education. While the provision of sexual health policies and training are important first steps towards caregivers' discussing SHR with LAC, both institutional factors and caregivers' own personal experiences and values can act as barriers to this occurring. In order for SHR discussions to become a routine aspect of caregiving, our results suggest that future revisions of SHR policy and training by social work departments should emphasise to caregivers the importance of collaborative working, the use of safe care procedures and the availability of managerial and pastoral support to help caregivers overcome these barriers and include SHR discussions within their practice. Emphasis also needs to be placed upon ensuring that inclusion of SHR within caregiving practices does not increase levels of caregiver strain or erode caregivers' sense of professional identify by creating role confusion. Further research is needed to ascertain whether caregiver-led SHR discussions improve sexual health outcomes for LAC.

For peer review only

For peer review only - http://bmjopen.bmj.com/site/about/guidelines.xhtml

Author contributions

CN designed the study, carried out the qualitative data collection and analysis, and drafted the manuscript. MH and LE participated in the design of the study and drafting the manuscript. All authors read and approved the final manuscript.

Funding

This work was supported by the UK Medical Research Council, the Scottish Government Chief Scientist Office, Edinburgh Napier University and Glasgow Caledonian University. CN as part of the Children, Young People, Families and Health and Social Relationships and Health Improvement Programmes [MC_UU_12017/2, MC_UU_12017/11 and SPHSU11], and MH as part of the Children, Young People, Families and Health, Social Relationships and Health Improvement, and Understanding and Improving Health within Settings and Organisations Programmes [MC_UU_12017/2, MC_UU_12017/11, MC_UU_12017/14 and SPHSU11] all at the MRC/CSO Social and Public Health Sciences Unit, University of Glasgow. CN was funded by a Scottish Government Chief Scientist Office Pre-doctoral Fellowship (CSO DTF/09/16). LE was core funded by the School of Nursing and Community Health, Glasgow Caledonian University. The funders had no role in study design, data collection and analysis, decision to publish, or preparation of the manuscript.

Acknowledgements

The authors would like to thank the Local Authority for their participation in this study, and all of the individuals who took part in the study and shared their thoughts and experiences. We would also like to thank Professor Lisa McDaid, Professor Daniel Wight and Dr Alice Maclachlan for commenting upon this manuscript.

Competing interests

The authors declare that they have no competing interests

For peer review only - http://bmjopen.bmj.com/site/about/guidelines.xhtml

Ethical approval and consent to participate

The College of Social Science Research Ethics Committee at the University of Glasgow reviewed and approved the study and consent procedure [Application number: SS/10/0027]. Study participants' provided written informed consent.

Data sharing agreement

No further data are available as this was not part of the ethics approval

For peer review only

For peer review only - http://bmjopen.bmj.com/site/about/guidelines.xhtml

Table 1: Perceived barriers and facilitators to inclusion of SHR discussions in the professional role categorised by implications for caregivers' understandings of their professional role

	Facilitator	*Barrier*
**Role ambiguity**	Provision of SHR policy	Lack of clear guidance on recording/reporting procedures
**Role conflict**	Development of strategies to monitor LAC sexual behaviour<br>Development of strategies to minimise risk of allegations<br>Having "parented" around sex<br>Own experiences of SHR education/discussions as a child	Competing responsibilities of managing risks, child protection concerns and undertaking preventative SHR discussions<br>Holding strong moral/religious attitudes about sexual behaviours
**Role overload**	Interagency working<br>SHR training<br>Support of management	Workforce capacity and composition<br>Not having sufficient time to build trusting relationships<br>Not having sufficient skills and knowledge

SHR, sexual health and relationships

For peer review only - http://bmjopen.bmj.com/site/about/guidelines.xhtml

References

1. *The Children and Young People (Scotland) Act 2014*. 2014, HMSO: London.
2. *Children Act*. 1989, HMSO: London.
3. The Scottish Government, *Children's Social Work Statistics 2015-16*. 2017, The Scottish Government: Edinburgh.
4. Directorate, I.A., *Children’s Social Care Statistics for NI 2016/17*. 2017, Department of Health: Stormont.
5. Government, W., *Children looked after by local authorities, 2016-17 (Experimental statistics)*. 2017.
6. Department of Education, *Children looked after in England including adoption: 2016 to 2017*. 2017, The Stationery Office: London.
7. The Scottish Government, *Children's Social Work Statistics Scotland, 2013-14*. 2015: Edinburgh.
8. Pecora, P.J., et al., *Mental health of current and former recipients of foster care: A review of recent studies in the USA.* Child & Family Social Work, 2009. **14**(2): p. 132-146.
9. Vinnerljung, B. and M. Sallnäs, *Into adulthood: a follow ‐up study of 718 young people who were placed in out ‐of ‐home care during their teens.* Child & Family Social Work, 2008. **13**(2): p. 144-155.
10. Hjern, A., F. Lindblad, and B. Vinnerljung, *Suicide, psychiatric illness, and social maladjustment in intercountry adoptees in Sweden: a cohort study.* The Lancet, 2002. **360**(9331): p. 443-448.
11. Vinnerljung, B., M. Öman, and T. Gunnarson, *Educational attainments of former child welfare clients–a Swedish national cohort study.* International Journal of Social Welfare, 2005. **14**(4): p. 265-276.
12. Dube, S.R., et al., *The impact of adverse childhood experiences on health problems: evidence from four birth cohorts dating back to 1900.* Preventive medicine, 2003. **37**(3): p. 268-277.
13. Bonell, C., et al., *The effect of dislike of school on risk of teenage pregnancy: testing of hypotheses using longitudinal data from a randomised trial of sex education.* Journal of Epidemiology and Community Health, 2005. **59**(3): p. 223-230.
14. Ellis, B.J., et al., *Does father absence place daughters at special risk for early sexual activity and teenage pregnancy?* Child Development, 2003. **74**(3): p. 801-821.
15. Ramrakha, S., et al., *Psychiatric disorders and risky sexual behaviour in young adulthood: cross sectional study in birth cohort.* BMJ, 2000. **321**(7256): p. 263-266.
16. Otto-Salaj, L.L., et al., *Psychiatric functioning and substance use: Factors associated with HIV risk among incarcerated adolescents.* Child Psychiatry and Human Development, 2002. **33**(2): p. 91-106.
17. Carpenter, S.C., et al., *The association of foster care or kinship care with adolescent sexual behavior and first pregnancy.* Pediatrics, 2001. **108**(3): p. e46-e46.
18. Ahrens, K.R., et al., *Laboratory-diagnosed sexually transmitted infections in former foster youth compared with peers.* Pediatrics, 2010. **126**(1): p. e97-e103.
19. Dworsky, A. and M.E. Courtney, *The risk of teenage pregnancy among transitioning foster youth: Implications for extending state care beyond age 18.* Children and Youth Services Review, 2010. **32**(10): p. 1351-1356.
20. Vinnerljung, B., E. Franzén, and M. Danielsson, *Teenage parenthood among child welfare clients: A Swedish national cohort study of prevalence and odds.* Journal of Adolescence, 2007. **30**(1): p. 97-116.
21. Henderson, M., et al., *Heterosexual risk behaviour among young teenagers in Scotland.* Journal of Adolescence, 2002. **25**(5): p. 483-494.

For peer review only - http://bmjopen.bmj.com/site/about/guidelines.xhtml

22. Ahrens, K.R., et al., *Psychosocial Pathways to Sexually Transmitted Infection Risk Among Youth Transitioning Out of Foster Care: Evidence From a Longitudinal Cohort Study.* Journal of Adolescent Health, 2013. **53**(4): p. 478-485.

23. Ahrens, K.R., et al., *Association between childhood sexual abuse and transactional sex in youth aging out of foster care.* Child Abuse & Neglect, 2012. **36**(1): p. 75-80.

24. Dale, H., et al., *The perceived sexual health needs of looked after young people: findings from a qualitative study led through a partnership between public health and health psychology.* Journal of Public Health, 2011. **33**(1): p. 86-92.

25. Knight, A., E. Chase, and P. Aggleton, *Teenage Pregnancy among Young People in and Leaving Care: Messages and Implications for Foster Care.* Adoption & Fostering, 2006. **30**(1): p. 58-69.

26. McFreely, S., *Teenage Pregnancy in Looked After Young People.* Practice Nurse

2003. **29**(37-42).

27. Love, L.T., et al., *Fostering hope: preventing teen pregnancy among youth in foster care.* Gender and Development, 2005. **13**(3): p. 34-41.

28. Constantine, W.L., P. Jerman, and N.A. Constantine, *Sex education and reproductive health needs of foster and transitioning youth in three California counties.* Public Health Institute. March, 2009. **2**.

29. Lipscombe, J., E. Farmer, and S. Moyers, *Parenting fostered adolescents: skills and strategies.* Child & Family Social Work, 2003. **8**(4): p. 243-255.

30. Bruce, J.S., *Sexual and reproductive health policies for foster youth in California: A qualitative study of child welfare professionals' experiences and perceptions of policies.* Children and Youth Services Review, 2016. **61**: p. 184-200.

31. Fullerton, D., et al., *Sexual Health and Sexuality Education Needs Assessment of Young People in Care in Ireland (SENYPIC): A Survey of Service-provider Perspectives. Report No. 1.* 2016, HSE Crisis Pregnancy Programme and the Child & Family Agency: Dublin.

32. Boonstra, H.D., *Teen pregnancy among young women in foster care: A primer.* Guttmacher Policy Review, 2011. **14**(2).

33. Geiger, J.M. and L.A. Schelbe, *Stopping the cycle of child abuse and neglect: A call to action to focus on pregnant and parenting youth in and aging out of the foster care system.* Journal of Public Child Welfare, 2014. **8**(1): p. 25-50.

34. Winter, V.R., R.A. Brandon-Friedman, and G.E. Ely, *Sexual health behaviors and outcomes among current and former foster youth: A review of the literature.* Children and Youth Services Review, 2016. **64**: p. 1-14.

35. DiClemente, R.J., et al., *Parent-adolescent communication and sexual risk behaviors among African American adolescent females.* The Journal of pediatrics, 2001. **139**(3): p. 407-412.

36. Stone, N. and R. Ingham, *Factors affecting British teenagers' contraceptive use at first intercourse: The importance of partner communication.* Perspectives on Sexual and Reproductive Health, 2002: p. 191-197.

37. Hutchinson, M.K., et al., *The role of mother–daughter sexual risk communication in reducing sexual risk behaviors among urban adolescent females: a prospective study.* Journal of adolescent health, 2003. **33**(2): p. 98-107.

38. DiIorio, C., E. Pluhar, and L. Belcher, *Parent-child communication about sexuality: A review of the literature from 1980–2002.* Journal of HIV/AIDS Prevention & Education for Adolescents & Children, 2003. **5**(3-4): p. 7-32.

39. Healthcare Policy and Strategy Directorate: Child and Maternal Health Division, *CEL-16: Implementation of action 15 of the looked after children and young people: we can and must do better report*. 2009, The Scottish Government,: Edinburgh.

40. The Scottish Government, *Better Health, Better Care: Action Plan*. 2007, HMSO: Edinburgh.

41. Stryker, S., *Symbolic interactionism: A social structural version*. 1980: Blackburn Press Caldwell,, NJ.

For peer review only - http://bmjopen.bmj.com/site/about/guidelines.xhtml

42. Stryker, S. and A. Statham, *Symbolic interaction and role theory*, in *The handbook of social psychology* G. Lindzey and E. Aronson, Editors. 1985, Random House: New York. p. 311-378.
43. Stryker, S. and R.T. Serpe, *Identity salience and psychological centrality: Equivalent, overlapping, or complementary concepts?* Social Psychology Quarterly, 1994: p. 16-35.
44. Brookes, K., et al., *Role theory: A framework to investigate the community nurse role in contemporary health care systems.* Contemporary Nurse, 2007. **25**(1-2): p. 146-155.
45. McKenna, H., S. Keeney, and M. Bradley, *Generic and specialist nursing roles in the community: an investigation of professional and lay views.* Health & social care in the community, 2003. **11**(6): p. 537-545.
46. Brown, B., P. Crawford, and J. Darongkamas, *Blurred roles and permeable boundaries: the experience of multidisciplinary working in community mental health.* Health & Social Care in the Community, 2000. **8**(6): p. 425-435.
47. Major, D.A., *Utilizing role theory to help employed parents cope with children's chronic illness.* Health education research, 2003. **18**(1): p. 45-57.
48. Gardner, J.S., et al., *Pharmacist prescribing of hormonal contraceptives: results of the Direct Access study.* Journal of the American Pharmacists Association: JAPhA, 2007. **48**(2): p. 212-21; 5 p following 221.
49. Austin, W., et al. *Moral distress in healthcare practice: The situation of nurses*. in *HEC Forum*. 2005. Springer.
50. Nixon, C., *Communicating about sexual health and relationships within local authority care placements.* 2015, University of Glasgow: Glasgow.
51. O'Brien, B., et al., *Standards for reporting qualitative research: a synthesis of recommendations.* Acad Med 2014. **89**(9): p. 1245-1251.
52. Gregory, A., et al., *School climate and implementation of a preventive intervention.* American journal of community psychology, 2007. **40**(3-4): p. 250-260.
53. The Scottish Government, *These are our bairns: a guide for community planning partnerships on being a good corporate parent*. 2008, Scottish Government Edinburgh.
54. East, L.J. and T.R. Orchard, *Somebody else's job: Experiences of sex education among health professionals, parents and adolescents with physical disabilities in Southwestern Ontario.* Sexuality and Disability, 2014. **32**(3): p. 335-350.
55. Sugerman, S., et al., *Family planning clinic patients: their usual health care providers, insurance status, and implications for managed care.* Journal of Adolescent Health, 2000. **27**(1): p. 25-33.
56. The Scottish Government, *Decision Making Guidance for Foster Carers*. 2015: Edinburgh.
57. Ruane, J. and M. Hayter, *Nurses' attitudes towards sexual relationships between patients in high security psychiatric hospitals in England: An exploratory qualitative study.* International Journal of Nursing Studies, 2008. **45**(12): p. 1731-1741.
58. Higgins, A., P. Barker, and C.M. Begley, *'Veiling sexualities': a grounded theory of mental health nurses responses to issues of sexuality.* Journal of Advanced Nursing, 2008. **62**(3): p. 307-317.
59. Pound, P., et al., *What is best practice in sex and relationship education? A synthesis of evidence, including stakeholders' views.* BMJ Open, 2017. **7**(5).
60. Farmer, E., J. Lipscombe, and S. Moyers, *Foster Carer Strain and its Impact on Parenting and Placement Outcomes for Adolescents.* The British Journal of Social Work, 2005. **35**(2): p. 237-253.
61. Dane, B., *Child Welfare Workers.* Journal of Social Work Education, 2000. **36**(1): p. 27-38.
62. Rhodes, K.W., et al., *Foster family resources, psychosocial functioning, and retention.* Social work research, 2003. **27**(3): p. 135-150.
63. Unrau, Y.A., J.R. Seita, and K.S. Putney, *Former foster youth remember multiple placement moves: A journey of loss and hope.* Children and Youth Services Review, 2008. **30**(11): p. 1256-1266.

For peer review only - http://bmjopen.bmj.com/site/about/guidelines.xhtml

64. James, S., et al., *Sexual risk behaviors among youth in the child welfare system.* Children and Youth Services Review, 2009. **31**(9): p. 990-1000.
65. Dvir, Y., et al., *Childhood maltreatment, emotional dysregulation, and psychiatric comorbidities.* Harvard review of psychiatry, 2014. **22**(3): p. 149.
66. Hoagwood, K.E., et al., *Family support in children's mental health: A review and synthesis.* Clinical child and family psychology review, 2010. **13**(1): p. 1-45.
67. Quinn, C., B. Happell, and G. Browne, *Talking or avoiding? Mental health nurses' views about discussing sexual health with consumers.* International journal of mental health nursing, 2011. **20**(1): p. 21-28.
68. Buston, K., et al., *Implementation of a teacher-delivered sex education programme: obstacles and facilitating factors.* Health Education Research, 2002. **17**(1): p. 59-72.
69. Faulder, G.S., et al., *Teaching sex education improves medical students' confidence in dealing with sexual health issues.* Contraception, 2004. **70**(2): p. 135-139.
70. Jaccard, J., P.J. Dittus, and V.V. Gordon, *Parent-Teen Communication about Premarital Sex Factors Associated With the Extent of Communication.* Journal of Adolescent Research, 2000. **15**(2): p. 187-208.
71. Elliott, L., C. Kennedy, and R. Raeside, *Professional role identity in shaping community nurses' reactions to nursing policy.* Journal of nursing management, 2015. **23**(4): p. 459-467.

For peer review only

For peer review only - http://bmjopen.bmj.com/site/about/guidelines.xhtml

SRQR checklist for manuscript: Providing sex and relationships education for looked after children: a qualitative exploration of how personal and institutional factors promote or limit the experience of role ambiguity conflict and overload among caregivers

		Reporting Item	Page Number
Problem formulation	#1	Concise description of the nature and topic of the study identifying the study as qualitative or indicating the approach (e.g. ethnography, grounded theory) or data collection methods (e.g. interview, focus group) is recommended	6
	#2	Summary of the key elements of the study using the abstract format of the intended publication; typically includes background, purpose, methods, results and conclusions	2
	#3	Description and signifcance of the problem / phenomenon studied: review of relevant theory and empirical work; problem statement	4-6
Purpose or research question	#4	Purpose of the study and specific objectives or questions	6
Qualitative approach and research paradigm	#5	Qualitative approach (e.g. ethnography, grounded theory, case study, phenomenolgy, narrative research) and guiding theory if appropriate; identifying the research paradigm (e.g. postpositivist, constructivist / interpretivist) is also recommended; rationale. The rationale should briefly discuss the justification for choosing that theory, approach, method or technique rather than other options available; the assumptions and limitations implicit in those choices and how those choices influence study conclusions and transferability. As appropriate the rationale for several items might be discussed together.	5-7
Researcher characteristics and reflexivity	#6	Researchers' characteristics that may influence the research, including personal attributes, qualifications / experience, relationship with participants, assumptions and / or presuppositions; potential or actual interaction between researchers' characteristics and the research questions, approach, methods, results and / or transferability	7
Context	#7	Setting / site and salient contextual factors; rationale	5, 7

For peer review only

		Reporting Item	Page Number
Sampling strategy	#8	How and why research participants, documents, or events were selected; criteria for deciding when no further sampling was necessary (e.g. sampling saturation); rationale	6-7
Ethical issues pertaining to human subjects	#9	Documentation of approval by an appropriate ethics review board and participant consent, or explanation for lack thereof; other confidentiality and data security issues	7, 24
Data collection methods	#10	Types of data collected; details of data collection procedures including (as appropriate) start and stop dates of data collection and analysis, iterative process, triangulation of sources / methods, and modification of procedures in response to evolving study findings; rationale	6-7
Data collection instruments and technologies	#11	Description of instruments (e.g. interview guides, questionnaires) and devices (e.g. audio recorders) used for data collection; if / how the instruments(s) changed over the course of the study	7
Units of study	#12	Number and relevant characteristics of participants, documents, or events included in the study; level of participation (could be reported in results)	6
Data processing	#13	Methods for processing data prior to and during analysis, including transcription, data entry, data management and security, verification of data integrity, data coding, and anonymisation / deidentification of excerpts	6-7
Data analysis	#14	Process by which inferences, themes, etc. were identified and developed, including the researchers involved in data analysis; usually references a specific paradigm or approach; rationale	7
Techniques to enhance trustworthiness	#15	Techniques to enhance trustworthiness and credibility of data analysis (e.g. member checking, audit trail, triangulation); rationale	7
Syntheses and interpretation	#16	Main findings (e.g. interpretations, inferences, and themes); might include development of a theory or model, or integration with prior research or theory	8-16
Links to empirical data	#17	Evidence (e.g. quotes, field notes, text excerpts, photographs) to substantiate analytic findings	8-16

For peer review only

		Reporting Item	Page Number
Intergration with prior work, implications, transferability and contribution(s) to the field	#18	Short summary of main findings; explanation of how findings and conclusions connect to, support, elaborate on, or challenge conclusions of earlier scholarship; discussion of scope of application / generalizability; identification of unique contributions(s) to scholarship in a discipline or field	16-19
Limitations	#19	Trustworthiness and limitations of findings	20-21
Conflicts of interest	#20	Potential sources of influence of perceived influence on study conduct and conclusions; how these were managed	23
Funding	#21	Sources of funding and other support; role of funders in data collection, interpretation and reporting	23

For peer review only

BMJ Open

Providing sex and relationships education for looked after children: a qualitative exploration of how personal and institutional factors promote or limit the experience of role ambiguity conflict and overload among caregivers

Journal:	*BMJ Open*
Manuscript ID	bmjopen-2018-025075.R1
Article Type:	Research
Date Submitted by the Author:	05-Feb-2019
Complete List of Authors:	Nixon, Catherine; University of Glasgow, MRC/CSO Social and Public Health Sciences Unit<br>Elliott, Lawrie; Glasgow Caledonian University, Department of Nursing and Community Health<br>Henderson, Marion ; University of Glasgow, MRC/CSO Social and Public Health Sciences Unit
**Primary Subject Heading**:	Sexual health
Secondary Subject Heading:	Qualitative research, Public health, Health policy
Keywords:	looked-after children, sexual health, caregivers, social workers, QUALITATIVE RESEARCH

SCHOLARONE™
Manuscripts

Providing sex and relationships education for looked after children: a qualitative exploration of how personal and institutional factors promote or limit the experience of role ambiguity conflict and overload among caregivers

Catherine Nixon[1*], Lawrie Elliott[2] and Marion Henderson[1]

For peer review only

Correspondence to:

Dr. Catherine Nixon

MRC/CSO Social and Public Health Sciences Unit, University of Glasgow, 200 Renfield Street, Glasgow, G2 3QB, Scotland, UK

catherine.nixon@glasgow.ac.uk

Keywords: looked-after children, sexual health, caregivers, social workers, qualitative research

For peer review only - http://bmjopen.bmj.com/site/about/guidelines.xhtml

Abstract

Objectives: To explore how personal and institutional factors promote or limit caregivers promoting sexual health and relationships (SHR) among looked-after children (LAC). In so doing, developing existing research dominated by atheoretical accounts of the facilitators and barriers of SHR promotion in care settings.

Design: Qualitative semi-structured interview study.

Setting: UK social services, residential children's homes and foster care.

Participants: 22 caregivers of LAC, including 9 foster carers, 8 residential carers and 5 social workers; half of whom had received SHR training.

Methods: In-depth interviews explored barriers/facilitators to SHR discussions, and how these shaped caregivers' experiences of discussing SHR with LAC. Data were systematically analysed using predetermined research questions and themes identified from reading transcripts. Role theory was used to explore caregivers' understandings of their role.

Results: SHR policies clarified role expectations and increased acceptability of discussing SHR. Training increased knowledge and confidence, and supported caregivers to reflect on how personally held values impacted practice. Identified training gaps were how to: 1) discuss SHR with LAC demonstrating problematic sexual behaviours and 2) record that SHR discussions had occurred in LAC's health plans.

Contrary to previous findings, caregivers regularly discussed SHR with LAC. Competing demands upon time resulted in prioritisation of discussions for sexually active LAC and those 'at risk' of sexual exploitation/harm. Interagency working addressed gaps in SHR provision. SHR discussions placed emotional burdens upon caregivers. Caregivers worried about allegations being made against them by LAC. Managerial/pastoral support and 'safe care' procedures minimised these harms.

Conclusions: While acknowledging the existing level of SHR promotion for LAC there is scope to more firmly embed this into the role of caregivers. Care needs to be taken to avoid role ambiguity and tension when doing so. Providing SHR policies and training,

For peer review only

promoting interagency working and providing pastoral support are important steps towards achieving this.

Article summary

Strengths and limitations of this study

- Looked after children are often excluded from school-based SHR education. The qualitative data generated from this study provides valuable insight into alternative means through which SHR discussions can be undertaken within this population.

  Participant accounts provided rich qualitative data that provided insight into how the introduction of SHR training and policies affected the undertaking of SHR discussions with looked-after children.

- Role theory underpinned the analysis and provided insight beyond existing research that is dominated by atheoretical accounts of the facilitators and barriers of SHR promotion in care settings.

- While our study focuses on the voices of residential care workers, foster carers and social workers it does not capture the experiences of kinship carers or parents whose children may be looked-after in their family home under the terms of a home supervision order.

For peer review only - http://bmjopen.bmj.com/site/about/guidelines.xhtml

Introduction

Children who are ‘looked-after’ are an important group for public health intervention. In the UK a child is considered to be a ‘looked-after child’ (LAC) if parental responsibility is assumed by social services or shared between the child’s parents and social services. LAC can experience a variety of care arrangements, including: foster care; placement in residential (children’s) homes, residential schools or secure units; being looked-after by relatives or family friends in kinship placements; and being looked-after at home with their parents under the supervision of social services [1, 2]. In 2016-2017, approximately 100,000 children were looked-after by local authorities in the four UK nations [3-6]. Whilst children may enter care as a result of their own or parental illness/disability, the majority become looked-after as a result of neglect or abuse [6, 7].

Childhood maltreatment and pre-care experiences characterised by poverty, family breakdown, domestic violence, and parental mental illness and/or substance abuse increase LAC’s vulnerability to experiencing: mental health difficulties; school exclusion; low educational attainment; substance use; and, antisocial/offending behaviour [8-11]. Cumulative exposure to these factors, which are independently associated with increased sexual risk-taking [12-16], mean LAC experience poorer sexual health outcomes than adolescents who have never been looked-after. For example, studies from the UK, USA and Sweden have demonstrated increased rates of early sexual activity, greater numbers of lifetime sexual partners, poorer contraceptive use, and increased rates of sexually transmitted infections (STIs), teenage pregnancy/parenthood, sexual exploitation/assault and rape [17-23].

The sexual health vulnerability of LAC may be exacerbated by limited access to SHR education. Barriers to SHR education include increased school absence/exclusion rates, frequent placement moves and poor-quality relationships with parents [24-26]. SHR discussions in care settings are also limited [24, 27-29]. For example, a survey of

For peer review only

For peer review only - http://bmjopen.bmj.com/site/about/guidelines.xhtml

Californian social workers reported that 66–77% had never spoken to LAC about puberty, the characteristics of healthy relationships, abstinence, contraception, sexual orientation, STIs, pregnancy/parenthood and abortion [28]. Reported barriers to caregivers discussing SHR include: lack of knowledge and training; difficulties reconciling personal religious/moral values; lack of guidance about who is responsible for discussing SHR with LAC; uncertainty as to whether such discussions infringe upon the rights of biological parents; concerns that discussing sex could be perceived as condoning sexual activity; uncertainty about the boundaries between confidentiality and child protection; and concerns about LAC making allegations of wrongdoing against caregivers [27, 28, 30, 31].

Calls have been made to improve LAC's access to SHR education [1, 32, 33]. Existing evidence shows that adolescents whose parents discuss SHR with them are more likely to delay intercourse, use contraception and have fewer sexual partners [34-37]. It has thus been recommended that caregivers discuss SHR with LAC. In Scotland, these recommendations have resulted in social work departments developing SHR policies and working with health-boards to provide training to caregivers [38, 39]. Key to this is changing the roles of those with legal responsibility for children in the care system.

To date, role theory [40-42] has been developed and applied in mainly health care settings. Research indicates that health practitioners develop specific beliefs and attitudes about their profession which establish the boundaries of their role and how they interact with other professionals, and those they serve [43]. Introducing new responsibilities can challenge these beliefs and blur the boundaries of the existing roles [44]. While role blurring has advantages, such as allowing practitioners to adopt new responsibilities and be more responsive to the needs of the population they are working with, it can result in them feeling overwhelmed and uncertain about the limits of their new role [45]. Role blurring can also introduce sources of conflict, additional workload and ambiguity to their professional role [43, 46]

For peer review only

Role conflict occurs when practitioners attempt to fulfil two or more roles with incompatible expectations, requirements, beliefs or attitudes. For example, healthcare practitioners with strong personal views about the acceptability of abortion may find it difficult to provide information or advice about terminating an unwanted pregnancy [47, 48]. Within the literature, role conflict is identified as a chronic source of stress that can result in healthcare professionals experiencing psychological distress [49]. Role overload occurs when the demands of a particular role exceed the capacity of the individual. For instance, through not having sufficient time to undertake tasks or through lacking the knowledge and skills necessary to undertake that role [43, 46]. Finally, role ambiguity occurs when there is a lack of guidance/clarity about roles, and can result in: practitioners being unclear about what is expected of them; create concerns about duplication of work; and, act as a source of frustration, tension or anxiety at work [46].

In this study we extend the application of role theory [40-42] to care settings, and more specifically, as a framework to answer the following research question: 'how do personal and institutional factors promote or limit the experience of role ambiguity, conflict and overload among caregivers tasked with discussing SHR with LAC?' In addressing this question we further develop the existing evidence base by moving beyond identifying barriers to SHR discussions being undertaken within the care system [24, 27, 29] and providing new insights into how SHR discussions can be incorporated into practice.

Methods

In-depth individual qualitative interviews were conducted with 22 caregivers from August-October 2011. As we wanted to explore how SHR discussions were influenced by caregivers' perceptions of their role, our sample included three groups of caregivers: foster carers (n=9), residential carers (n=8) and social workers (n=5).

For peer review only - http://bmjopen.bmj.com/site/about/guidelines.xhtml
For peer review only

Recruitment and sample characteristics

All caregivers were recruited from a large urbanised local authority in Scotland, which had recently introduced SHR training for caregivers. In order to minimise service disruption, it was agreed with the local authority that sampling of social workers would be restricted to those based within 1 of the 3 geographically-based teams providing care to LAC. Sampling of foster carers was restricted to foster carers supervised by social workers working within that team. Residential carers were not geographically recruited; however, in order to minimise disruption it was agreed that unit managers, rather than frontline residential care staff, would be approached.

Respondents were randomly selected from staff lists stratified by whether caregivers had received SHR training or not. Our aim was to interview caregivers when half of all foster carers, residential carers and social workers had received training as it was felt that doing so would more accurately capture the role that training had upon the inclusion of SHR discussions for each of the different caregiving roles. However, institutional delays in rolling out training meant that interviews were undertaken when half the foster carers, all residential carers and no social workers had received training. Our achieved sample reflects this.

The caregivers recruited were predominantly female (n=19); reflecting the gender imbalance seen within social services in the UK [50]. Participants' age was not formally recorded; however we know from ethnographic notes describing participants that foster carers were generally older (estimated age range of mid-forties to mid-sixties) than residential carers and social workers (estimated age range early thirties to mid-fifties). At the time of interview, the number of children living in placement was 1-3 for foster carers and 4-8 for residential carers. Information about average caseloads among social workers was not obtained, although we know from remarks made by one social worker during interview that she was managing 34 cases aged 0-16. In 2009 a freedom of information request reported the average caseload among social workers was 7 [51].

For peer review only - http://bmjopen.bmj.com/site/about/guidelines.xhtml
For peer review only

Interview process

Interviews focussed on: SHR training; experiences of providing advice about SHR; perceived barriers/facilitators to undertaking SHR discussions; which SHR topics were discussed; and how discussions were shaped by the experiences of LAC and their caregivers. A copy of the interview schedule can be found in Supplementary File 1.

Interviews were undertaken by the lead author (CN). Foster carers were interviewed within family homes without children and young people present, while residential carers and social workers were interviewed within private offices in residential children's homes and social work offices. Participants were not compensated for their time at the request of the local authority; however, they were afforded time within their working day to participate in interviews. On average, interviews lasted 45-60 minutes. Interviews were digitally recorded and transcribed verbatim.

Data analysis and management

Prior to analysis, transcripts were compared with original recordings to ensure that narratives had been accurately captured, with any mistakes or missing segments updated by CN prior to coding being undertaken. Fieldwork notes on body language, facial expressions and emotional responses to questions were added to transcripts to supplement textual meaning. Transcripts were then anonymised and entered into NVivo 9 (QSR International, Melbourne, Vic., Australia) for data management and coding.

Thematic analysis was used to analyse the interview data generated. An initial coding framework was developed by CN based on both predetermined research questions and themes identified through close reading of all interview transcripts. The co-authors of this paper (MH and LE) reviewed the initial coding framework, along with a selection of transcripts, to identify any additional themes that they felt were missing from the initial coding frame. As part of this process, co-authors identified theories that they felt could be used to further refine the coding framework, with role theory [40-42] identified as a

For peer review only - http://bmjopen.bmj.com/site/about/guidelines.xhtml

potential means of understanding how introducing SHR provision into the professional role impacted upon caregivers construction of their professional and personal identities.

The coding frame was revised by CN to reflect caregivers' experiences of role ambiguity, conflict and overload. Themes were also coded according to whether they acted as barriers or facilitators to the inclusion of SHR discussions within the professional role. At this stage, deletion and consolidation of themes was also undertaken. The finalised coding framework was agreed by all authors, before being applied to all manuscripts by CN. This allowed systematic comparisons to be made across the data and facilitated the identification of recurrent themes. During the coding process it was agreed by the authors that data saturation was occurring, with similar descriptions and narratives being presented by participants. Thus, further data collection was deemed unnecessary.

Ethical approval

Ethical approval was granted by Glasgow University's Faculty of Law, Business and Social Sciences Ethics Board [Application number: SS/10/0027]. The research tools were also reviewed and approved by the Director of Children and Family Services on behalf of the local authority. SRQR reporting guidelines [52] were used to oversee the design, analysis and reporting of findings; details per Supplementary File 2.

Patient involvement

Patients were not involved in the design of this study.

Results

Our results highlight that inclusion of SHR discussions within caregiving is influenced by the interplay of institutional factors and caregivers' personal values/experiences. Table 1 provides a thematic overview of these factors and how they promoted or limited SHR promotion by contributing to the experience of role ambiguity, conflict and overload

For peer review only

among caregivers. Illustrative quotes, referred to by number (e.g. A1), are presented within both the main body of results and Table 1 as supportive evidence of the themes discussed.

Provision of sexual health policy and training reduces role ambiguity

Role ambiguity occurs when there is a lack of guidance and clarity about roles, and can result in practitioners being unclear about what is expected of them [46]. In this study we found that the provision of SHR policies reduced role ambiguity and increased the acceptability of SHR discussions among residential carers and social workers. This occurred by creating an institutional-level ethos that challenged historical taboos about discussing sex and emphasised the inclusion of SHR discussions within caregivers' professional role. For instance: *"We have policies that we follow now in terms of sexual health and it's something that's been brought to the forefront, where it's no considered taboo" (A1 - Shona, social worker/relief residential carer).*

In particular, SHR policies emphasised that undertaking SHR discussions with LAC allowed caregivers to fulfil their "corporate parenting" responsibilities: *"you would talk to your kids about it (sex). And that's what we do as corporate parents. We take on that role and responsibility" (A2 - Rachel, social worker).* Among residential carers and social workers, incorporating institutional expectations about corporate parenting responsibilities into practice resulted in their developing personally held views about what it meant to be a "good" corporate parent. These views focussed upon how as caregivers they should ensure that LAC were provided with the same access to SHR information as children not in the care system. Hence, SHR discussions were identified as being part of routine caregiving (A3).

The importance of corporate parenting was not reported by foster carers, who instead discussed their personal views that SHR discussions were part of normal family life: *"we don't shy away. If there was a sex scene in a movie or whatever, we quite often discuss*

For peer review only - http://bmjopen.bmj.com/site/about/guidelines.xhtml

For peer review only

it rather than say 'oh my goodness, we shouldn't be watching that, hide your eyes boys!' We just relax about it. It's not something that we try to pretend isn't there" (A4- Alison, foster carer).

SHR policies and training clarified at an institutional level what was expected of caregivers when discussing SHR with LAC. For instance, one caregiver described how the individual within social work services had come along to a staff meeting to discuss the policy and the role that residential carers were expected to play in implementing it: *"Jane [policy developer] came to the unit manager's meeting and was kind of promoting young people's sexual health, what was our responsibility and where did we see our responsibilities being. And the training was very informative. It was very informative and made us look at our own sexual health and relationships. It gave us the tools to go away and… have these discussions wi' young people" (A5 - Patricia, residential carer).* SHR training was also identified as clarifying the range of topics that caregivers would be expected to discuss with LAC. This clarification was welcomed by several residential carers who had been reprimanded in the past for doing things such as accompanying LAC to obtain emergency contraception (e.g. A5 – Joanne, residential carer).

Lack of guidance contributes to role ambiguity

While on the whole SHR policies reduced role ambiguity, lack of institutional guidance on specific pract elements created confusion contributed to role ambiguity. One example of this was how SHR discussions should be recorded, with residential carers perceiving that they did not have clear guidance about how to confidentially record and present information when there were no child protection concerns. For instance, one residential carer stated: *"recording it is very difficult. We have general comms [communication] books which are for everybody's viewing which is not appropriate, and we have individual logs which aren't appropriate either because the kid is maybe keen to keep something in confidence but then it is written down. It is a grey area" (A6 – Mark).*

For peer review only - http://bmjopen.bmj.com/site/about/guidelines.xhtml

Uncertainty about how to record information in a way that protected the confidentiality of LAC was perceived as a potential barrier to LAC approaching caregivers for help.

Role conflict

Role conflict occurs when practitioners attempt to fulfil two or more roles with incompatible expectations, requirements, beliefs or attitudes [47, 48]. In this study we identified three examples of how including SHR interactions into caregiving practices resulted in three sources of role conflict that acted as barriers to SHR. These were: 1) the challenges of balancing the competing responsibilities of child protection concerns with undertaking preventative SHR discussions; 2) caregivers' concerns about the potential for allegations being made against them by LAC influencing decisions about how and when SHR discussions were undertaken; and 3) challenges that caregivers faced in reconciling professional responsibilities for discussing SHR with personally held religious/moral views about sexual behaviours. These challenges, and the approaches that caregivers used to address them, will now be discussed.

Balancing competing demands of child protection and preventative SHR work

Caregivers experienced role conflict as a result of trying to balance the competing institutional responsibilities of child protection concerns with undertaking preventative SHR discussions. Many of those interviewed stated that their role often focussed more on policing/monitoring sexual behaviour than providing SHR information: *"I was no longer a caregiver – I was a security guard" (B1 - Karen, foster carer/former residential carer).* As the main institutional priority was to safeguard LAC, focussing on risk-taking meant that SHR discussions were often undertaken solely in response to specific safety concerns: *"there wouldn't always be a major, in-depth discussion if there weren't any major issues… but if a child is sexually active, and they're underage, and… running away, having sex wi' [with] men they don't know, coming back the next morning covered in mud, drinking… it would be very high on the agenda" (B2 - Agnes, social worker).* This resulted in some LAC being excluded from SHR discussions.

For peer review only - http://bmjopen.bmj.com/site/about/guidelines.xhtml

The challenges of managing child protection risks were particularly emphasised by those caring for LAC who had experienced sexual exploitation/abuse and/or were demonstrating sexually inappropriate or sexually abusive behaviours towards others. Caregivers identified a range of strategies advocated by their institution to manage the sexual health of LAC, including: monitoring peer and romantic relationships (B3); monitoring mobile phone and computer usage (B4); assessing the risks associated with young people in their care going on both supervised and unsupervised outings (B5); and carefully managing internal public spaces and room allocations (B6). For example, one residential carer described how the space within residential care settings was carefully managed to provide additional monitoring and to protect residents: *"We've got one young person who… can display quite predatory behaviour [later clarified by the caregiver stating that the young woman had been groomed into recruiting other LAC for a sex ring]. She would encourage the rest of the group to go out drinking, and then make allegations of rape against one or more of the boys... We need to protect her and we need to protect others from her exposing them to inappropriate sexual contact for their age. That's something that we balance all the times in terms of the safety of the group. And that's how we decided her bedroom was best placed in close relation to the office" (B6 – Joanne).*

Having strategies to draw upon appeared to reduce the experience of role conflict for caregivers by situating some SHR discussions within the context of risk management (e.g. B4 – Claire).

Role conflict as a source of caregiver strain

Although institutional supports were available for managing the sexual health of LAC, the constant monitoring of behaviour understandably affected caregivers' emotional wellbeing. For example, one foster carer became upset and started visibly shaking while recounting an incident where she had found two siblings who had been left unsupervised *"in the sliding wardrobe, a pillow put down on the inside to mask the noise... and the girl*

For peer review only - http://bmjopen.bmj.com/site/about/guidelines.xhtml

on the floor as he was on top of her holding her down" (B7 – Jean). The perceived impacts of caregivers' emotional distress on SHR provision was not directly discussed.

Concerns about the potential for false allegations being made by LAC

Being asked to discuss SHR with LAC was perceived as a task that placed caregivers in a position of vulnerability. For instance, one of the social workers described a situation where being left alone with a young man who had been groomed and sexually abused by a paedophile ring had left her line manager feeling very vulnerable: *"I had to leave the room and when I came back my manager was like 'I needed to come out' and basically he'd been sitting and the boy had got an erection. He felt really uncomfortable cos obviously he was on his own with him and he didn't want to be on his own with him… so he got up and walked out. As workers we can be quite vulnerable" (C1 – Agnes).*

Feeling vulnerable resulted in caregivers expressing concerns that undertaking SHR discussions could result in false allegations of abuse being made against them by LAC. These concerns contributed to the experience of role conflict by highlighting to caregivers the potentially negative reputational and financial consequences that could occur if steps weren't taken to reduce the risk of allegations occurring: *"It's worrying… my son's a police officer, my husband works in law enforcement and I work with students – so given that we all have to be vetted and disclosed at work - we have to take extra care" (C1 - Alison, foster carer).*

Wanting to protect against false allegations affected how SHR discussions were undertaken, with all of those interviewed emphasising how following institutional guidelines and developing 'safe care' practices helped minimise the risk of allegations being made against caregivers. The practices adopted by caregivers included ensuring that discussions were accurately documented (C2), having conversations in private spaces with another caregiver present (C3) and developing household rules about things like personal contact, privacy and modesty (C4) These practices were also used to

safeguard children and other family members living within the household (e.g. C4 – Karen).

Personal values and experiences

The institutional expectation that SHR should be discussed with LAC contributed to the experience of role conflict among caregivers who held religious and moral views about the appropriateness of openly discussing sexual behaviours. For instance, one of the foster carers described how her faith had impacted upon views about her practice as a caregiver: *"I've got a Catholic upbringing and you didn't do anything until you were married. It was very strict. I wouldn't force that [talking about sex] on any of the kids that I work with" (D1 – Anne-Marie).*

Being provided with time in SHR training sessions to reflect upon how personal values could affect SHR discussions was identified as one way that the institution could support caregivers in addressing this conflict: *"It has took me an awful long time tae do all my challenging in myself and asking and prying aboot [about] how does that fit with my psyche to sit here and talk about things that I ordinarily would not talk aboot" (D2 - Claire, residential carer).* Pastoral support was also identified as an institutional means of supporting caregivers experiencing difficulties reconciling personal views with professional expectations. For example, one residential carer described supporting a fellow caregiver who strongly opposed abortion to *"present all the options"* to a girl in her care who wanted to terminate the pregnancy (D3 – Joanne).

Although religious and moral views acted as a source of conflict for some caregivers, other experiences acted to reduce role conflict by providing caregivers with personal motivation for incorporating SHR discussions into their professional practice. For example, caregivers' own experiences of not receiving school- or home-based SHR education as a child reduced conflict by providing motivation for SHR discussions being incorporated into practice: *"I went to college at sixteen and… I'm sitting in a class and*

For peer review only - http://bmjopen.bmj.com/site/about/guidelines.xhtml

I'm looking at this film on childbirth and I see where a baby's born from. I thought that they untied your tummy button, took it out, tied it up again and stuck it back in. Now I did bring my children up... from when they were wee tots… I would get them to go and get my sanitary towels and I would tell them what it was" (D4 - Pat, foster carer). Having discussed SHR with a biological child also helped to reduce conflict by providing caregivers with experiences that they could draw upon when talking to LAC: *"it was always just a natural kind of growing up. We spoke about contraception, and my daughter, I was able to go with her to the doctors when she wanted to start taking the pill. We could just talk about it really openly. Likewise, with Michael [foster child], we've approached the subject of puberty and changes in the body" (D5 - Alison, foster carer).*

Role overload

Role overload occurs when the demands of a particular role exceed the capacity of the individual. For instance, through not having sufficient time to undertake tasks or through lacking the knowledge and skills necessary to undertake that role [43, 46]. Lack of capacity acted as a barrier to SHR discussions being undertaken by caregivers and was associated with the experience of role overload. Four instances of this were identified: 1) workforce capacity i.e. not having sufficient time to discuss SHR with LAC; 2) workforce composition; 3) not having the necessary skills/knowledge to talk about sexual health; and 4) needing managerial and pastoral support to undertake SHR discussions. These barriers, along with the strategies used to address these, will now be discussed.

Workforce capacity

Having insufficient numbers of caregivers acted as source of role overload and acted as an institutional barrier to residential carers' undertaking SHR discussions. For instance, one residential carer described the challenges that limited staffing presented in supporting a young man in placement who had both *"been sexually abuse*d" and *"sexually abused younger children"* to provide appropriate opportunities for socialisation, monitor his behaviours and discuss the effects of previous abuse on his understandings

For peer review only - http://bmjopen.bmj.com/site/about/guidelines.xhtml

of relationships: *"We're limited wi' [with] staff. We should have two on every shift so if you had a member of the team who was doing that work maybe 2-3 hours a week there is an impact on the other five young people you're looking after" (E1 – Patricia).* The number of children living in placement was identified as contributing to role overload, with larger group home settings presenting more challenges to staff (e.g. E1 – Joanne).

Social workers also reported having limited capacity to discuss SHR with LAC. The main reason cited for this was that competing demands meant social workers did not have time to build up the trusting relationships LAC formed with residential carers, foster carers and keyworkers provided by external agencies: *"as a social worker it's a lot more difficult to really get to know that young person because in residential… you really get to know the young people because you see them for 24-hour periods, and you know a lot more about their life, and what's happening on a daily basis… being a social worker… there's a lot more hidden. You maybe find out a month later that something happened… and it's a lot more difficult to establish what. Spending time wi' young people and building up that relationship is what opens more doors to the speaking to you directly about it [SHR]" (E2 - Lindsay, social worker).* Institutional demands upon social workers time, along with the perception that families distrusted social workers, led to it being perceived that the role of the social worker should focus upon coordinating and monitoring the promotion of SHR to LAC: *"a safety plan gets planned and implemented… and focused work carried out that is specific and tailored to that young person's needs and risks… that's something that as the allocated worker I would review and monitor" (E2 - Mary, social worker).*

To ensure that LAC were not excluded from SHR discussions due to limited capacity among caregivers to undertake these, all of those interviewed stressed the importance of inter-agency and partnership working. For instance, Patricia (residential carer) returned to her example of the young man who had been "sexually abused" and "sexually abused younger children" to describe how several agencies worked to support

For peer review only - http://bmjopen.bmj.com/site/about/guidelines.xhtml

caregivers to monitor his behaviour and undertake SHR discussions with him: *"It's a bit aboot [about] sharing you know? We kind of all come together. Agency X does the risk assessment work, and they work wi' the young person about why it happened, their feelings, whatever. Agency Y work wi' him to provide socialisation – taking him out because obviously he's not allowed out unsupervised" (E3).* This was particularly valued when the SHR topics that needed to be discussed with LAC related to more specialised topics such as sexual exploitation and the adoption of problematic sexual behaviours which caregivers did not have sufficient time or training to focus upon (e.g. E3 – Joanne).

Partnership working had the potential to contribute to role overload by duplicating the workloads of caregivers. To avoid this, and to ensure LAC did not receive contradictory messages, caregivers highlighted the importance of being informed about the content of SHR education that was delivered by partner organisations: *"we've had reports from them and we know what they are doing… so we tend to back off and let one person do that work on sexual health and keeping safe" (E4 - Joanne, residential carer).*

Workforce composition

The low proportion of male residential carers was identified as a potential barrier to male LAC receiving SHR information: *"If there wasnae a male on shift then the boys wouldn't come and talk to us about sex" (E5 - Laura, residential carer).* There were two reasons for this. First, it was perceived by caregivers that LAC were more comfortable and less embarrassed discussing SHR with caregivers of the same sex (E6). Second, female caregivers perceived that male caregivers, as a result of their lived experiences, were often better placed to explain or demonstrate sex-specific hygiene practices: *"teaching them how to shave for example, that's not something I can do. So, if I have a male worker, then I get him to come into work unshaven so he can show the boys how to shave properly" (E7 - Trisha, residential carer).* Not having sufficient numbers of men working in residential care was identified as a potential source of role overload for female

For peer review only - http://bmjopen.bmj.com/site/about/guidelines.xhtml

residential carers as a result of their having to take on the role of being predominantly responsible for undertaking SHR discussions.

Not having sufficient skills and knowledge

Not having the skills and training needed to undertake SHR discussions contributed to the experience of role overload and acted as a barrier to such discussions being undertaken by caregivers. Being provided with SHR training by social work services was identified as a way to address this: *"if I was in the position of working with a young person who had a very trusting relationship with me, and who required support with their sexual health and development, then I would like to play a part in that… but I'd like training because I see that as a gap" (F1 - Mike, social worker).*

SHR training was reported to improve caregivers' knowledge of sexual health terminology, contraceptive choices, STIs and colloquialisms used by LAC to describe sexual acts: *"the course opens your eyes to it, you know? You can go through life thinking, well, right, ok, I know about Gonorrhoea and this kind of stuff, but I don't know about Chlamydia, and I don't know about this, that and the next thing. And these are all things that children can get, and I need to be able to explain what can happen if they have unprotected sexual relationships" (F2 - Ian, foster carer).* This reduce role overload by providing caregivers with the specialist SHR knowledge that they reported was needed to incorporate SHR discussions into practice. Having more specialised SHR knowledge was also perceived by caregivers as a means of ensuring that LAC were provided with information that was factually correct, not confusing and unable to be misconstrued: *"one worker talked about your flower, and if you needed anything sorted you would go to the flower shop… I don't think that things like that really help when talking about going to clinics and your vulva. You need to use the proper names so that everyone is quite clear as she could have people thinking 'oh right, I need to go buy some flowers'… because they take you literally" (F2 - Laura, residential carer).*

For peer review only - http://bmjopen.bmj.com/site/about/guidelines.xhtml

Being able to draw upon SHR knowledge improved caregivers' confidence and reduced embarrassment. In turn, this facilitated discussions of SHR with LAC. Caregivers who had received SHR training highlighted the importance of being provided with leaflets and books that they could use to help discuss SHR with LAC: *"after the training I found that I was really more confident and I had all the information on hand and booklets to show" (F3 - Maria, foster carer).* Caregivers also discussed how the training had provided them with details of websites and helplines that they could use if they were asked questions that they did not know the answer to. Having these resources was valued as it allowed caregivers to help answer questions they might not have known the answer to: *"If I've no got an answer for them I'll maybe say 'we've got literature on that so just gie me a minute and we'll go and get it and we'll take five minutes to go through it" (F4 - Patricia, residential carer).* Training resources were particularly valued for helping caregivers tailor SHR discussions to the physical, emotional and cognitive ages of LAC.

While SHR training provided caregivers with the skills needed to incorporate SHR discussions into practice, it should be noted that all of the residential carers and foster carers, regardless of whether they had received SHR training or not, routinely discussed SHR with LAC. The range of topics reported as being discussed within caregivers' narratives included puberty, peer relationships, romantic relationships, masturbation, having sex for the first time, contraception, reproduction and sexual exploitation. The main difference observed was that caregivers who had not received SHR training were more likely to express personal concerns about the accuracy of the information they had provided: *"we were playing Connect 4, and one of the girls said 'how do you get pregnant' and we said 'well, you need to have sex'. 'Aye, I know that… and I know that he cums, but how does that then work?' So we dismantled the Connect 4, and we said 'well it's no square, but you'll have to imagine this is a womb, and these are the fallopian tubes', and we used the wee circles as the sperms and the eggs, and we used that to explain it…. Once we were finished I turned to [another caregiver] and said 'did I get that right?'" (F5 - Claire, residential carer).* These concerns further emphasised the

For peer review only - http://bmjopen.bmj.com/site/about/guidelines.xhtml

For peer review only

important role that SHR training played in reducing role overload by bolstering caregivers' knowledge and confidence.

Pastoral support

Pastoral support from managers and other caregivers helped caregivers who felt unable to talk about sexual health because of insufficient knowledge or training engage in SHR discussions. This was particularly valued when caregivers had to discuss SHR with LAC who had been sexually abused, groomed or exploited and were demonstrating problematic sexual behaviours: *"I found that work really difficult, because I had never had to deal with… trying to manage a child – cos he was a child at the time — who is not only, you know, being abused, but is an abuser… I felt really, you know, unsure of how best to manage that. One of the best things with managing that was that my manager agreed to support me, and we did the work together" (Agnes, social worker).*

Being supported by managers to talk to LAC about SHR reduced role overload. The main reason for this being that caregivers supported to undertake discussions that they found to be challenging and did not have the skills and training needed to undertake these discussions alone. Peer networks also reduced role overload by providing caregivers with a group of individuals that they could use as source of help and advice: *"We deal with it pretty well, but I think with this wee core group of carers that we've got there's always an opportunity for learning… 'I've tried to get this boy to do his bloody homework and he just will not do it' and somebody will say 'try this' and you find that it works. That's where our support is… from other carers in our group. We bounce off each other" (G2 – Ian, foster carer).*

Discussion

Using role theory [40-42] we have identified that the introduction of SHR discussions into caregiving practices for LAC can result in caregivers experiencing role ambiguity,

For peer review only - http://bmjopen.bmj.com/site/about/guidelines.xhtml

conflict and overload. Providing SHR policies and training, and encouraging caregivers to develop strategies that safeguarded all individuals living within placement were identified as ways in which institutions, in this case social work services, could work to reduce role ambiguity and conflict. Our results also highlights the important role that the institution can play in minimising caregivers' experience of role overload specifically related to the incorporation of SHR discussions into practice, namely through the provision of SHR training, provision of pastoral supports and encouraging the use of inter-agency working. Contrary to the results of previous studies, which have shown that SHR discussions within LAC settings are limited [24, 27, 29], our results demonstrate that caregivers regularly discussed SHR with LAC. These findings further develop existing research that is dominated by atheoretical accounts of the facilitators and barriers of SHR promotion in care settings.

Institutional support for intervention delivery contributes to the successful adoption of general health prevention programmes [53]. Our results support this finding by demonstrating that caregivers incorporate discussing SHR with LAC into their usual caregiving practices when social work departments invest in creating an organisational culture that reduces taboos about sex and clearly outlines what is expected of caregivers. In this study, culture change was underpinned by corporate parenting policy which states that all caregivers have a legal duty to support LAC to obtain the "*same health outcomes… as any good parent would want for their own children*" [54]. This appealed to caregivers desire to be viewed as being 'good' parents to the children in their care; suggesting that the framing of SHR policies to reflect the professional identity of caregivers can act as an important first step towards the successful incorporation of SHR discussions into practice.

Lack of guidance about who should be responsible for discussing SHR is known to act as a barrier to LAC receiving SHR information and advice in non-LAC settings [27, 28, 30, 31]. Our results show that introducing SHR policies in LAC settings reduced this

For peer review only - http://bmjopen.bmj.com/site/about/guidelines.xhtml

ambiguity by clarifying role expectations and emphasising that talking to LAC about SHR was the responsibility of all caregivers. This is important as previous studies in non-LAC settings have demonstrated that when role ambiguity occurs, both parents and health professionals choose not to discuss SHR with adolescents due to perceptions that there are others who are better qualified to undertake this role [55].

While on the whole our results show that policy can be used to clarify the role that caregivers can play in discussing SHR with LAC, our results demonstrated that caregivers required additional guidance on how to confidentially record SHR discussions. Lack of confidentiality is a known barrier to adolescents approaching services for SHR information [56]. Thus, caregivers not being able to record information in a confidential manner has the potential to prohibit LAC from engaging in open SHR discussions with caregivers. To minimise the risk of this happening, we recommend that SHR policies and training should provide clear guidance on how and where caregivers should record SHR discussions, and outline the circumstances under which information sharing is required, namely where child protection concerns exist [57]. This is particularly important in residential settings, where multiple caregivers require access to LAC's health information.

In this study, some of the caregivers interviewed discussed how they had drawn upon their personal experiences of discussing SHR with their biological children when undertaking SHR discussions with LAC. While having personal experiences to draw upon may facilitate SHR discussions, evidence from secondary schools shows that in the absence of SHR guidance and training, teachers incorporate their own moralistic views of sexual behaviour into SHR discussions with pupils [58, 59]. Among our sample, moralistic views were identified as a reason for choosing not to discuss SHR with LAC or opting out of providing information on sensitive issues such as abortion; a finding echoed in other studies looking at healthcare practitioners' attitudes towards contraception and carrying out medical terminations of pregnancy [47, 48]. As it is known that adolescents

For peer review only

For peer review only - http://bmjopen.bmj.com/site/about/guidelines.xhtml

do not seek SHR information from adults they perceive to be judgemental [60] it is important that caregivers are trained to provide factual, evidence-based information about SHR.

SHR policies and training should take into account the burdens that caring for LAC place upon caregivers. Our results highlight that the time demands associated with managing child protection concerns often resulted in SHR discussions not being undertaken unless support from external agencies was sought or the role of meeting the sexual health needs of LAC was undertaken by specialist public health (LAC) nurses. The caregivers interviewed in this study had lots of experience with multi-agency working, and their experiences highlight the need for clear delineation of roles when discussing SHR with LAC. To that end, the health plans of LAC should clearly outline: their sexual health needs; what information/intervention is required; and which individuals/agencies will be involved in meeting those needs and how. A lead professional should be identified and tasked with the responsibility of overseeing any multi-agency involvement, and ensuring that all individuals/agencies involved have clearly delineated roles and responsibilities [30]. This is particularly important for LAC at risk of sexual exploitation or demonstrating problematic sexual behaviours.

Our results highlighted caring for LAC who had experienced sexual abuse/exploitation had emotional impacts upon caregivers. Caring for young people demonstrating risky behaviours can result in caregivers experiencing vicarious trauma and strain [61, 62]. Identifying ways to minimise caregiving strain is important as it is known that this can contribute to placement breakdowns [61, 63]. Repeated breakdowns of placements are known to erode the ability of LAC to form secure and trusting relationships with their caregivers [64], which in turn may lead to LAC being unable to identify a consistent and trusted adult that they can discuss SHR with [25, 28, 65, 66]. Thereby further excluding LAC from accessing SHR information and advice.

For peer review only - http://bmjopen.bmj.com/site/about/guidelines.xhtml

Caregiving strain can negatively impact upon the sensitivity of caregivers' parenting skills, particularly the ability to respond to the emotional rather than physical ages of children [61]. As the majority of LAC have experienced child maltreatment [6], known associations between maltreatment, emotional dysregulation and cognitive delay [67] increase the likelihood that caregivers will need to tailor preventative SHR messages to the emotional and cognitive ages of LAC. Working to alleviate caregiving strain is thus an important step to ensuring that age-appropriate SHR discussions are undertaken with LAC. One potential way of alleviating caregiving strain is through peer-led support services. For instance, recent evidence shows that the provision of peer support for parents with children experiencing mental health difficulties reduces isolation, increases social support, empowers caregivers and increases levels of self-care [68]. Future research should explore whether peer-led sexual health support services for caregivers can empower caregivers to undertake SHR discussions, while reducing caregiving strain.

Finally, our results emphasise the importance of training for facilitating SHR discussions within the care setting. SHR training providing caregivers with the confidence, knowledge and skills needed to undertake SHR discussions with LAC. These findings reflect results from other studies of sexual health, which have demonstrated that the introduction of SHR training improves the confidence of teachers and medical professionals to talk with pupils and patients about SHR [69-71] and increases the likelihood of parents engaging in conversations about sex with adolescents [72]. It is encouraging that training facilitated SHR discussions, as evidence shows that adolescents whose parents discuss SHR with them are more likely to delay intercourse, use contraception and have fewer sexual partners [34-37]. Further research should establish whether caregiver-led SHR discussions can contribute to a reduction in sexual risk among LAC.

For peer review only - http://bmjopen.bmj.com/site/about/guidelines.xhtml

Reflections on study conduct

Feminist research practices challenge the view that research can and should be value free [73], and advocates that researchers should reflect upon how personal characteristics of both the research team and participants may have influenced participation, engagement and the credibility of findings [74]. Here, we briefly reflect upon how who we were as individuals and the choices that we made as a research team affected the research process. In doing so we identify some of the methodological limitations and strengths of this study.

Looking first at recruitment and data collection, we are aware that our decision to access caregivers through social work services could result in some caregivers feeling pressured into participating. As we were aware that there was an expectation among senior managers that caregivers 'would' participate we made sure that as researchers we took preventative steps to ensure that caregivers were fully informed of their rights, i.e. that they could choose not participate in the research, could choose how much information they wanted to disclose and that they could withdraw from the study at any point without us informing their employers that they had done so. On the whole, we feel that these steps led to caregivers feeling that they had the right not to participate, with several of those contacted declining to participant. However, one of the caregivers that CN interviewed appeared to be participating out of expectation rather than choice. The data collected from this interview was very closed, lacked the same depth of information that other caregiver interviews contained and was terminated early.

Recruiting caregivers through social work services also raised the issues of power-dynamics and bias within the research process. For example, the social work department that we recruited participants through acted as a gatekeeper to participants and senior managers played an active role in identifying the area-based team that social workers would be recruited from and choosing which residential carers would be contacted. From the perspective of senior management the decisions that they made were designed to

For peer review only - http://bmjopen.bmj.com/site/about/guidelines.xhtml

minimise any disruptions to service associated with caregivers participating in research; however, it is also possible that these decisions were made to ensure that caregivers who were known to be good at discussing SHR with LAC were interviewed. Thus, casting both the social work department and the SHR training that they had introduced in a good light. To counter this possibility, it was agreed that caregivers would be randomly selected from staff lists.

While there was potential for biased narratives to be collected, our analysis of data identified that caregivers were often quite critical of social work services, the training that they had received and the practices of other caregivers. In many ways, the fact that none of the research team had histories of being in care, or providing care to LAC, meant that we were often viewed as naïve outsiders who needed to be educated about the care system and participant's experiences of caring for LAC. The data generated could have been very different if our interviewer (CN) had had any connections to social work services. For instance, it is possible that the data collected would have been less detailed as a result of participants not fully elaborating on practices, policy and training due to the perception that the researcher already had access to this information.

Finally, it should be noted that our decision to use role theory as a means of interpreting our findings partly reflected the research interests of LE, who was involved in another study exploring how professional role identity shaped the reactions of community nurses to changes in nursing policy. The identification that the themes being coded could be mapped to the definitions of role ambiguity, role conflict and role overload was viewed as a strength by us due to the existing literature relating to the sexual health of LAC being largely atheoretical. Had LE not been familiar with role theory and its application with health settings it is possible that other theoretical lenses would have been used to interpret our research findings; resulting in a potentially different interpretation of caregiver narratives.

For peer review only - http://bmjopen.bmj.com/site/about/guidelines.xhtml

Strengths and limitations

Strengths of this qualitative interview study include the generation of rich qualitative data that provided insight into how the introduction of SHR training and policies facilitated SHR discussions within the care setting, and how caregivers negotiated and addressed perceived impacts of undertaking SHR discussions upon their professional and personal identities. As LAC are often excluded from school-based SHR education, the data generated from this study provides valuable insights into alternative means through which SHR discussions can be undertaken within this population. Role theory underpinned the analysis and provided insight beyond existing research that is dominated by atheoretical accounts of the facilitators and barriers of SHR promotion in care settings.

This study has two limitations. First, and most importantly, our study does not capture the experiences of kinship carers or parents who children may be looked-after at home under home supervision orders. Future research should explore how the experiences of these caregivers differs from those in our study who are more professionally oriented, and in particular, the extent to which our understanding of role theory applies. Second, while our aim was to interview caregivers when half of all foster carers, residential carers and social workers had received SHR training, institutional delays in rolling out training to caregivers meant that interviews occurred when half the foster carers, all residential carers and no social workers had received training. While our achieved sample reflects this, it is possible that our findings for social workers in particular do not fully capture the impact of policy that encourages discussion about SHR with LAC. It would be beneficial if future revisions of sexual health training and policy within the care system reflected evaluative work being undertaken in the field of nursing [75] and collected pre- and post- quantitative and qualitative data on the effect that introducing new policy has upon the constructions of caregivers' professional identity.

For peer review only - http://bmjopen.bmj.com/site/about/guidelines.xhtml

Conclusion

To encourage SHR discussions within the care setting it is important that organisations adopt a culture that is supportive of LAC receiving SHR education. While the provision of sexual health policies and training are important first steps towards caregivers' discussing SHR with LAC, both institutional factors and caregivers' own personal experiences and values can act as barriers to this occurring. In order for SHR discussions to become a routine aspect of caregiving, our results suggest that future revisions of SHR policy and training by social work departments should emphasise to caregivers the importance of collaborative working, the use of safe care procedures and the availability of managerial and pastoral support to help caregivers overcome these barriers and include SHR discussions within their practice. Emphasis also needs to be placed upon ensuring that inclusion of SHR within caregiving practices does not increase levels of caregiver strain or erode caregivers' sense of professional identify by creating role confusion. Further research is needed to ascertain whether caregiver-led SHR discussions improve caregivers' professional identity and reduce the sexual vulnerability of LAC.

For peer review only

For peer review only - http://bmjopen.bmj.com/site/about/guidelines.xhtml

Author contributions

CN designed the study, carried out the qualitative data collection and analysis, and drafted the manuscript. MH and LE participated in the design of the study and drafting the manuscript. All authors read and approved the final manuscript.

Funding

This work was supported by the UK Medical Research Council, the Scottish Government Chief Scientist Office, Edinburgh Napier University and Glasgow Caledonian University. CN as part of the Children, Young People, Families and Health and Social Relationships and Health Improvement Programmes [MC_UU_12017/2, MC_UU_12017/11 and SPHSU11], and MH as part of the Children, Young People, Families and Health, Social Relationships and Health Improvement, and Understanding and Improving Health within Settings and Organisations Programmes [MC_UU_12017/2, MC_UU_12017/11, MC_UU_12017/14 and SPHSU11] all at the MRC/CSO Social and Public Health Sciences Unit, University of Glasgow. CN was funded by a Scottish Government Chief Scientist Office Pre-doctoral Fellowship (CSO DTF/09/16). LE was core funded by the School of Nursing and Community Health, Glasgow Caledonian University. The funders had no role in study design, data collection and analysis, decision to publish, or preparation of the manuscript.

Acknowledgements

The authors would like to thank the Local Authority for their participation in this study, and all of the individuals who took part in the study and shared their thoughts and experiences. We would also like to thank Professor Lisa McDaid, Professor Daniel Wight and Dr Alice Maclachlan for commenting upon this manuscript.

Competing interests

The authors declare that they have no competing interests

For peer review only - http://bmjopen.bmj.com/site/about/guidelines.xhtml

Ethical approval and consent to participate

The College of Social Science Research Ethics Committee at the University of Glasgow reviewed and approved the study and consent procedure [Application number: SS/10/0027]. Study participants' provided written informed consent.

Data sharing agreement

No further data are available as this was not part of the ethics approval.

For peer review only

For peer review only - http://bmjopen.bmj.com/site/about/guidelines.xhtml

827 **Table 1: Qualitative mapping of how institutional and personal factors act as barriers/facilitators of SHRE discussions and**
828 **contribute to role ambiguity, conflict and overload among caregivers.**

	**Institutional or Personal level factor**	**Barrier/ Facilitator of SHR discussions**	**Effect upon caregiving role**
**ROLE AMBIGUITY**			
Provision of sexual health policy and training reduces role ambiguity			
A1. Challenges perceived taboos about discussing sex<br>*"We have policies that we follow now in terms of sexual health, and it's something that's been brought to the forefront, where it's no considered taboo" (Shona, social worker/relief residential carer)*<br><br>*"In this industry, social services, there's been a lot of taboo about discussing sexual health. It's a priority now and it's seen as part and parcel of anything. I think that's cos of the policy and training" (Mary, social worker)*<br><br>*"It frees it up. You feel that, aye, it's no this big thing that shouldnae [shouldn't] be talked about. Children need to learn. They need to know that and we need to stop making it this big thing that they need to thing out themselves" (Pat, foster carer)*	Institutional	Facilitated SHR discussions by providing guidance and training	Reduced role ambiguity by emphasising that caregivers could and should discuss SHR with LAC
A2. Emphasises corporate parenting responsibilities<br>*"We are corporate parents and we would do it with our own kids" (Joanne, residential carer).*<br><br>*"You would talk to your kids about it [sex]. And that's what we do as corporate parents. We take on that role and responsibility" (Rachel, social worker).*<br><br>*"If we don't discuss it with a child, I think educate is too strict a term, but if we don't make them aware of it, then how are they gonnae [going to] know? (Shona, social worker/relief residential carer).*	Institutional	Facilitated SHR discussions by providing guidance and training	Reduced role ambiguity by clarifying expectations around caregiving role
A3. What it means to be a "good" corporate parent<br>*"We have a corporate parenting responsibility to all our kids but the key word there is parenting. Any good parent would spend time with their children talking about what is appropriate, when it is appropriate and how they should find out more information. As a*	Personal	Facilitated SHR by promoting personal involvement in professional task	Reduced role ambiguity as a result of policy focus reflecting

parent you are trying to encourage young people to discuss with you that they've got partners, that they are engaged in sexual relationships, that they have got a girlfriend or boyfriend or whatever it may be… It's about reassuring young people that I am asking the exact same questions I ask my own daughter or my own son. You aren't being treated any differently because you live in a unit" (Mark, residential carer).			personal beliefs about parenting
A4. Policy reflects what usually happens within families<br>*"We don't shy away. If there was a sex scene in a movie or whatever, we quite often discuss it rather than say 'oh my goodness, we shouldn't be watching that, hide your eyes boys!' We just relax about it. It's not something that we try to pretend isn't there" (Alison, foster carer)*<br><br>*"The real conversations we have in here at tea times– and it always starts off with something silly. I had… was it a banana and custard yoghurt I had yesterday? And that started it off – one of them says: 'oh, that looks terrible' and this girl says 'but you can't determine whether you like it or not by looking at it. You've got to taste it'. 'No, I don't'. And this taste thing went on and on and it got into a discussion of peer pressure, didn't it? How it got there, I don't know. We just sit at the table through there and talk" (Ian, foster carer)*	Personal	No perceived effect reported by caregivers	No effect
A5. Clarifies expectations of role<br>*"Jane [policy developer] came to the unit manager's meeting and was kind of promoting young people's sexual health, what was our responsibility and where did we see our responsibilities being. And the training was very informative. It was very informative and made us look at our own sexual health and relationships. It gave us the tools to go away and… have these discussions wi' [with] young people" (Patricia, residential carer).*<br><br>*"We've never had any form of policy or training towards sexual health. It's something that as a manager I can say to them 'you've been given the information, you've been given the tools to deal with that situation, you have to put it into practice now" (Mark, residential carer).*<br><br>*"The training was mainly sharing stories about sexual health. You know, would you get a young person the morning after pill and what is age appropriate for that? I think we've progressed. A few years ago I was on holiday with a young person and I took her for the morning after pill and I had my bum booted… 'You shouldn't have done that. It wasn't your decision to take. Fortunately we've moved on as a department'" (Joanne, residential carer).*	Institutional	Facilitated SHR discussions by providing guidance and training	Reduced role ambiguity by clarifying expectations around caregiving role

For peer review only

For peer review only - http://bmjopen.bmj.com/site/about/guidelines.xhtml

Lack of guidance contributes to role ambiguity<br><br>A6. Lack of clear guidance on recording/reporting procedures<br>*Recording it is very difficult. We have general comms [communication] books which are for everybody's viewing, which is not appropriate, and we have individual logs which aren't appropriate either because the kid is maybe keen to keep something in confidence but then it is written down. It is a grey area" (Mark, residential carer).*<br><br>*"I had a LAC review where there young person, there was issues in terms of she was menstruating and leaving dirty sanitary towels and pants, like planting them places and hiding them. So I had written the report and I made a comment about some hygiene issues and said to my manager beforehand. There was a reason I had made it really vague as I didn't want to embarrass her. Unfortunately the foster carer decided to start talking about it and the girl burst into tears… and what I suppose I'm trying to highlight is that we need to be sensitive to the young person (Agnes, social worker).*	Institutional	Perceived barrier to LAC approaching caregivers for help and advice	Contributed to role ambiguity by creating confusion about how best to confidentially record SHR discussions
**ROLE CONFLICT**<br><br>***Balancing competing demands of child protection and preventative SHR work***<br>B1. Monitoring sexual behaviour acts as a barrier to undertaking SHR discussions<br>*"Safety is paramount" (Jane, residential carer)*<br><br>*"I was no longer a caregiver – I was a security guard. Keeping young ones out of other one's rooms that weren't supposed to be there, hauling other ones in windows that were trying to get out in the middle of the night, keeping ones out that didn't belong to the unit. We had fifteen year olds that we were hauling out of one room into another and saying 'No. You're not on'" (Karen, foster carer/former residential carer).*	Institutional	Barrier to preventative SHR discussions being undertaken due to focus on risk management	Contributed to role conflict
B2. Undertaking SHR discussions in response to risk rather than preventatively<br>*"There wouldn't always be a major, in-depth discussion if there weren't any major issues… but if a child is sexually active and they're underage, and… running away, having sex wi' men they don't know, coming back the next morning covered in mud, drinking… it would be very high on the agenda" (Agnes, social worker).*<br><br>*"He'd put on his profile something like 'I'm in care and I'm a gay boy and I'm looking for…' One of the older girls had seen his profile and asked him right out in front of us 'why have you got that one your profile'. He was mortified. But that gave us the opportunity to sit down*	Institutional	Barrier to preventative SHR discussions being undertaken due to focus on risk management<br><br>Facilitator of SHR discussions in	Contributed to role conflict

For peer review only - http://bmjopen.bmj.com/site/about/guidelines.xhtml

and tell him the reasons why he should have things like that on there. And even if you are gay, it's not the way you would word it, and it was actually our 16 year old who said 'cos you don't know who is sitting looking at that profile and thinking oh he's game'" (Joanne, residential carer)		response to risk-taking by LAC	
B3: Strategies to manage the sexual health of LAC: monitoring relationships<br>*"We have a young female (16) who is pregnant and her boyfriend (23) lives locally. He had been over for dinner and he has been involved in the unit and staff have met him and we are clear what our role is. It was quite clear to us that the best way for us to deal with it was to be part of the relationship. I was quite clear that in my role of safeguarding this young girl we had to get to know this young male and find out if there was any ulterior motive or if there was any reasons why he was interested in her, other than you know, a love for each other. So we engaged with him... We have been to his house on a couple of occasions, and we have met with his mum as well. (Mark, residential carer)*<br><br>*"You are trying to encourage the young people to discuss with you that they've got partners and to bring them in so that we know them as a face round the unit. They're not allowed in bedrooms obviously, but they're allowed in the living area with the door open. And I would definitely encourage that unless I thought it was a negative influence" (Joanne, residential carer)*	Institutional	Facilitated SHR discussions around appropriate and positive relationships	Reduced role conflict
B4: Strategies to manage the sexual health of LAC: monitoring phone and computer usage<br>*"I'm no' that good at it, but we went into his facebook and realised the chats he's been having so we've started to speak about safety issues, you know, telling him that this person could be roond [round] the corner fae [from] you. It's a web cam" (Claire, residential carer).*<br><br>*"There was inappropriate material found on her phone, and in the past she's had images sent to her from people that in my opinion are grooming her, but she doesn't accept that she's at risk... So now we've got monitoring sheets. We monitor every shift what kids are doing on the computer and sometimes we think it's a wee bit of an overkill and obviously our internet is kind of sitting in the living room, very open, but we keep a very very close eye... especially when you think they are at risk" (Joanne, residential carer).*	Institutional	Facilitated SHR discussions around internet safety and sexual exploitation	Reduced role conflict
B5: Strategies to manage the sexual health of LAC: risk assessing outings<br>*"Last summer we stopped taking him to the play park… because he goes to younger children and he wants to pat them and cuddle them. I don't know if he is sexually aware… but he is almost compelled to do it… and he will sneak about to try and get to a wee one to give them*	Institutional	Barrier to preventative SHR discussions being undertaken due to	Reduced role conflict

For peer review only - http://bmjopen.bmj.com/site/about/guidelines.xhtml

a wee pat. So how do you deal with that? We stop taking him" (Pat, foster carer)<br><br>*"If you've got child protection issues where you've got a young person who's maybe been sexually abused, and then sexually abused younger people, then we have to be dead strict as protecting other young people is also protecting them… I cannae [cannot] let him run aboot [about] just down the road because there's a wee nursery doon [down] the road. I cannae just let him go swimming. There's a whole protection risk assessment to which there" (Patricia, residential carer)*		focus on risk management	
B6: Strategies to manage the sexual health of LAC: managing space and room allocations<br>*"We had a serious incident where Craig (13) accused John (8) of more or less sexually abusing him. John was saying things like 'sex, sex, sex' and making thrusting movements because he knew it was upsetting Craig…. Craig couldn't deal with it. We found him urinating on John's bed and then he made this accusation. It was a terrible time for us all, only for it to turn out that Craig had made the whole thing up… as he wanted John moved. So we're very aware now of the two boys being separate. Craig sleeps upstairs and he has his own space up there. John is downstairs in a room along the corridor, and he is not allowed upstairs at all" (Alison, foster carer)*<br><br>*"We've got one young person who most definitely has been sexually abused… and she can display quite predatory behaviour [later clarified by the caregiver stating that the young woman had been groomed into recruiting other LAC for a sex ring]. She would encourage the rest of the group to go out drinking, and then make allegations of rape against one or more of the boys... We need to protect her and we need to protect others from her exposing them to inappropriate sexual contact for their age. That's something that we balance all the times in terms of the safety of the group. And that's how we decided her bedroom was best placed in close relation to the office" (Joanne, residential carer)*	Institutional	Facilitated SHR discussions around privacy	Reduced role conflict
Role conflict as a source of caregiver strain<br>B7: Emotional impacts on caregivers<br>*"It's soul-destroying tae [to] try and stop that pattern of behaviour where young people would go met their pals… and be picked up by men that were pimping them… for a packet of cigarettes or a wee bag of sweeties. There would be times when they didnae [did not] want to have sex but they were forced and they would come in wi' pretty bad bruising and faces had been punched… It's pretty hard at times, but I think you've got tae be and be professional and say 'we're trying our best… sometimes we just don't succeed" (Patricia, residential carer)*	Personal	No perceived effect reported by caregivers	Consequence of role conflict

For peer review only - http://bmjopen.bmj.com/site/about/guidelines.xhtml

"I had been away shopping and I came back in. The other boy was watching television and he seen me and goes 'I think you should go up the stairs'. Now this has happened on a few occasion, you know? If one of us has been out or distracted they would use that moment. I just put my bag down, didn't even take my coat off, and I ran up the stairs [Jean is visibly shaking and obviously upset]. Here was child A and B in the sliding wardrobe, a pillow put down on the inside of the sliding wardrobe. He had the girl on the floor and he was on top of her… that's how quickly" (Jean, foster carer)			
**Concerns about the potential for false allegations being made by LAC**			
C1 Discussing SHR places caregivers in a position of vulnerability<br>*"I had to leave the room and when I came back my manager was like 'I needed to come out' and basically he'd been sitting and the boy [who had been groomed and sexually abused by a paedophile ring] had got an erection. He felt really uncomfortable cos obviously he was on his own with him and he didn't want to be on his own with him… so he got up and walked out. As workers we can be quite vulnerable… so we have to be very aware of how we protect ourselves (Agnes, social worker).*<br><br>*"You imagine right that one of the young persons' approached you right and said 'I'm thinking o' having sex wi' my boyfriend. What do you think? And then the next night the nightshift comes on and you're away and they say 'guess what she was saying tae me last night. Aw she was doing was talking about sex'. That can be misconstrued and before you know it it's a big fact finding investigation" (Patricia, residential carer)*<br><br>*It's worrying… my son's a police officer, my husband works in law enforcement and I work with students – so given that we all have to be vetted and disclosed at work - we have to take extra care". (Alison, foster carer).*	Personal	Barrier to SHR discussions arising from caregivers' concerns about their own vulnerability	Contributed to role conflict
C2 Strategies to protect caregivers against false allegations: recording conversations<br>*"Because of the risk that it presents to them as workers in terms of possible allegations or comments being made in future… we need to make sure that any information we are sharing with young people is appropriately recorded, accurately recorded… And if there is anything inappropriate, you know I am thinking, you know, maybe a female resident making a comment to a male member of staff then that's been appropriately recorded and raised and that the staff member and the young person are both supported and discussions are held about what is appropriate and what is not" (Mark, residential carer)*	Institutional	Facilitated SHR discussions by providing a safer environment for caregivers	Reduced role conflict

For peer review only - http://bmjopen.bmj.com/site/about/guidelines.xhtml

"I think that one of the things we had to obviously highlight was Safe Care and the recording of that sensitive conversation… how do you have that conversation in an environment where you're safe? Because if you're talking about closed doors she could make an allegation against you. So it's aboot recording the discussion you had, You don't have tae dae War and Peace but 'she came and she asked me aboot this and this was the advice I gave her'" (Patricia, residential carer).			
C3: Strategies to protect caregivers against false allegations: having someone else present<br>*"John had a wee urine infection and his penis was so sore, so it was a case of 'well, let's have a wee look and see if it's all red'… he's comfortable with that and it's all fine, but as a foster carer I'm not gonna go into a room and close a door and have a look at a 10 year old's penis. I'm gonna say 'right, Mark [husband] and I will sit on the bed and you touch it. You show me' and then 'right, ok, here's some cream' (Alison, foster carer)*<br><br>*"You would wait until the house was quieter and maybe do some of that work. 'Why don't we go on the computer next door and we'll shut the dining room door over' but I'll have a member of staff going in and out of the kitchen' (Anna, residential carer)*	Institutional	Facilitated SHR discussions by providing a safer environment for caregivers	Reduced role conflict
C4: Strategies to protect caregivers against false allegations: household rules<br>*"You need to keep reinforcing what is and is not appropriate behaviour… it is not appropriate to be showing yourself off. It's not appropriate to be going into the toilet with other boys" (Karen, foster carer)*<br><br>*"The wee things that you don't actually think about change, because, you know, it was quite natural for our boys to come down in the morning in their boxer shorts – maybe wae a dressing gown in the summer, maybe not. That changes. That stops. All that stops. You, you have to look at all the risks there are, and your, your children's life changes. Our seven-year-old couldn't come and jump into our bed in the morning because I couldn't allow the other two children to do it – so I couldn't allow him to do it because I didn't want them to feel that he was special" (Pat, foster carer)*	Institutional	Facilitated SHR discussions about privacy and boundaries	Reduced role conflict
**Personal values and experiences**			
D1 Religious and moral values as a source of role conflict<br>*"I'm a practising Catholic. I don't hold the church in any great high esteem but I have faith and as a parent myself I have never brought my children up tae… all this input of you can go get the pill here, you can go get a jag here and here's what all that's about in such graphic detail. I know this sounds as if its' so traditional and old fashioned but I was never brought*	Personal	Barrier to SHR discussions, particularly those focussed upon sex, sexuality and	Contributed to role conflict

For peer review only - http://bmjopen.bmj.com/site/about/guidelines.xhtml

	up with all this input. I suppose I'm still traditional in my own family home" (Claire, residential carer)<br><br>*"I've got a Catholic upbringing and you didn't do anything until you were married. It was very strict. I wouldn't force that [talking about sex] on any of the kids that I work with" (Anne-Marie, foster carer)*<br><br>*"Faith based values among staff can sometimes act as a barrier to workers discussing sexual health with young people" (Joanne, residential care)*		abortion	
D2	Being allowed to challenge and reflect upon values in training<br>*"It has took me an awful long time tae do all my challenging in myself and asking and prying aboot [about] how does that fit with my psyche to sit here and talk about things that I ordinarily would not talk aboot. I went on that course and I found it so challenging. 'Why are we talking about sex to these weans? Why are we no' talking about relationships?' And I got in this pure big debate wi' myself: 'I wouldnae tell my boy that. I wouldnae tell my lassies that' and the trainer was really helpful with me and saying 'yeah, but you need to remember that these kids arenae getting' what your own kids are getting'" (Claire, residential carer).*<br><br>*"There can be a clash between what workers may want and what the city council wants…so the training was very much to do with looking at our values, our value base and our knowledge… what was very surprising was the fact that kdis are learning so much younger, we were like 'oh my goodness, kids are talking about that [sex] at such a young age'" (Laura, residential carer)*	Institutional	Facilitated SHR discussions by challenging pre-existing beliefs and emphasising vulnerability of LAC	Reduced role conflict
D3	Pastoral support as a means of supporting caregivers experiencing role conflict<br>*"One of the foster carers I work with, she's never been used to talking to children about sex in any way and she asked me to undertake that as I had went on the training" (Anne-Marie, foster carer)*<br><br>*"She was really struggling with the fact that one of the girls in her care had approached her and told her that she was pregnant, but wanted to terminate the pregnancy. She was Catholic and very uncomfortable. My view was that this worker already had a relationship with this girl so it was my job to support her to present all the options to her". (Joanne, residential care)*	Institutional	Facilitated SHR discussions by providing caregivers with support to discuss challenging topics OR through providing LAC with access to another caregiver to discuss issues with	Reduced role conflict

D4 Own experiences of sexual health and relationships as a motivator for discussing SHR<br>*"I went to college at sixteen and… I'm sitting in a class and I'm looking at this film on childbirth and I see where a baby's born from. I thought that they untied your tummy button, took it out, tied it up again and stuck it back in. Now I did bring my children up... from when they were wee tots… I would get them to go and get my sanitary towels and I would tell them what it was" (Pat, foster carer)*<br><br>*"We got told nothing, absolutely nothing, to the stage where the first time I took a period I thought I was dying. And then when I had my first baby I didn't have a clue what was happening or what was going to happen to me so I always thought that if I had children of my own I would prepare them (Anne-Marie, foster carer)*	Personal	Facilitated SHR discussions by motivating caregivers to ensure that LAC received better access to information than they had during childhood	Reduced role conflict
D5 Having "parented" around sex<br>*"It was always just a natural kind of growing up. We spoke about contraception, and my daughter, I was able to go with her to the doctors when she wanted to start taking the pill. We could just talk about it really openly. Likewise, with John [foster child], we've approached the subject of puberty and changes in the body" (Alison, foster carer).*	Personal	Facilitated SHR discussions by providing caregivers with parenting experiences to draw upon	Reduced role conflict
**ROLE OVERLOAD**			
**Workforce capacity**<br>E1 Limited staff numbers in residential care<br>*"We're limited wi' [with] staff. We should have two on every shift so if you had a member of the team who was doing that work maybe 2-3 hours a week there is an impact on the other five young people you're looking after" (Patricia, residential carer)*<br><br>*"Children's unit staff are really well-placed to do stuff like that [discuss SHR]. They should be able to spend the time, but sometimes it doesn't seem to happen. I don't know why. I don't know if they're caught up in paperwork and ordering things, and dealing with incidents that have happened" (Louise, social worker)*<br><br>*"I worked in a 19-23 bedded unit and it was, the work was mostly chaotic. It was like firefighting and you were just going in and trying to contain your shift" (Joanne, residential carer)*	Institutional	Barrier to SHR discussions due focus on risk management and having to prioritise resources	Contributed to role overload

E2 Competing demands upon social workers time<br>*"I don't think we have the time to give young people the time they need and the support they need. That's just the way things are going to be. The service is just getting narrower… Sometimes you don't even have time to go to training as you get called to court" (Agnes, social worker)*<br><br>*"As a social worker it's a lot more difficult to really get to know that young person because in residential… you really get to know the young people because you see them for 24-hour periods, and you know a lot more about their life, and what's happening on a daily basis… being a social worker… there's a lot more hidden. You maybe find out a month later that something happened… and it's a lot more difficult to establish what. Spending time wi' young people and building up that relationship is what opens more doors to the speaking to you directly about it [SHR]" (Shona, social worker/relief residential carer).*<br><br>*"a safety plan gets planned and implemented… and focused work carried out that is specific and tailored to that young person's needs and risks… that's something that as the allocated worker I would review and monitor" (Mary, social worker).*	Institutional	Barrier to SHR discussions due to caregivers having limited time to form trusting relationships with LAC	Contributed to role overload
E3 The importance of interagency working to ensure that LAC receive SHR<br>*"We've got a 12 year old girl… and all her talk and her chat is about paedophiles, and she was going on websites and there was inappropriate material found on phones so obviously our alarm bells are ringing... She's so vulnerable. We've still not got feedback from the police what was on the phone. We give her wee trust exercises back on the computer but then she just tries to go onto these certain websites. She's had images sent to her from people that in my opinion are grooming her and she doesn't see that, she doesn't accept that she's at risk… So, we spoke to her worker at the young woman's project and she's covering a lot of that groundwork with her. And someone here is doing the work about keeping herself safe and making safer choices on the computer" (Joanne, residential carer)*<br><br>*"It's a bit aboot [about] sharing you know? We kind of all come together. Agency X does the risk assessment work, and they work wi' the young person about why it happened, their feelings, whatever. Agency Y work wi' him to provide socialisation – taking him out because obviously he's not allowed out unsupervised" (Patricia, residential carer)*	Institutional	Facilitator SHR discussions by providing additional supports to undertake concentrated SHR work	Reduced role overload
E4 Avoiding duplication of workload<br>*"We've had reports from them and we know what they are doing… so we tend to back off and let one person do that work on sexual health and keeping safe" (Joanne, residential*	Institutional	No perceived effect reported by caregivers	Reduced role overload

For peer review only - http://bmjopen.bmj.com/site/about/guidelines.xhtml

carer).<br><br>*"Sharing of information is key" (Mary, social worker)*			
**Workforce composition**			
E5. Low proportion of men working in residential care excludes male LAC from SHR discussions<br>*"There's no gender balance in residential… for every hundred applicants I can guarantee you that about 84% of them are women" (Patricia, residential carer)*<br><br>*"If there wasnae a male on shift then the boys wouldn't come and talk to us about sex" (Laura, residential carer)*<br><br>*"I dinnae really think boys have really come and speak to ye as much as girls, but then again, they might be more likely tae speak tae like a male, like a male worker" (Shona, social worker/relief residential carer)*	Institutional	Barrier to male LAC accessing SHR information	Contributed to role overload for female caregivers
E7 Male caregivers better placed to talk to LAC males about sex-specific practices<br>*"Teaching them how to shave for example, that's not something I can do. So, if I have a male worker, then I get him to come into work unshaven so he can show the boys how to shave properly" (Trisha, residential carer)*<br><br>*"He was always pulling at himself, wasn't he? And I said, 'do you know something? You need, when you're in the shower, you need to get your penis, pull your foreskin back and clean it with soap and water'. And he just stood there, but it cured it, didn't it?" (Ian, foster carer)*	Personal	Facilitated male LAC accessing SHR information when male caregivers were available to discuss issues	Reduced role overload for female caregivers
**Not having sufficient skills and knowledge**			
F1 Caregivers identifying that they need specialist training to undertake SHR discussions<br>*"if I was in the position of working with a young person who had a very trusting relationship with me, and who required support with their sexual health and development, then I would like to play a part in that… but I'd like training because I see that as a gap" (Mike, social worker).*<br><br>*"For workers, especially for workers who are not used to working with teenagers there is a need for more formal training, and formal training more often… I mean we do refresher*	Personal	Barrier to discussing SHR with LAC due to caregivers' perceived lack of knowledge about SHR topics and how to discuss these with LAC	Contributed to role overload

For peer review only - http://bmjopen.bmj.com/site/about/guidelines.xhtml

	courses for other training, but I can't remember the last time I saw a sexual health awareness or sexual health program (Agnes, social worker).			
F2	Sexual health and relationships training as a source of knowledge<br>*"A lot of the training was about words you've not heard since you were a kid… we need to know what these kids mean when they are saying certain things" (Joanne, residential carer)*<br><br>*"the course opens your eyes to it, you know? You can go through life thinking, well, right, ok, I know about Gonorrhoea and this kind of stuff, but I don't know about Chlamydia, and I don't know about this, that and the next thing. And these are all things that children can get, and I need to be able to explain what can happen if they have unprotected sexual relationships" (Ian, foster carer).*<br><br>*"one worker talked about your flower, and if you needed anything sorted you would go to the flower shop… I don't think that things like that really help when talking about going to clinics and your vulva… You need to use the proper names so that everyone is quite clear she could have people thinking 'oh right, I need to go buy some flowers'… because they take you literally" (Laura, residential care)*	Personal	Facilitated SHR discussions by providing caregivers with SHR knowledge and the skills needed to discuss these with LAC	Reduced role overload
F3	Sexual health and relationships training as a source of confidence<br>*"After I went to the training I found that I was really more confident and I had all the information on hand and booklets to show to the boy... and he said to me at the very end that he'd been having sex education at school, but that I had explained it far better. I put that down to the training" (Anne-Marie, foster carer)*<br><br>*"the training has definitely equipped the staff with confidence" (Tricia, residential carer)*	Personal	Facilitated SHR discussions by promoting confidence and reducing embarrassment among caregivers	Reduced role overload
F4	Using sexual health promotion materials<br>*"If I've no got an answer for them I'll maybe say 'we've got literature on that so just gie me a minute and we'll go and get it and we'll take five minutes to go through it" (Patricia, residential carer).*<br><br>*"to be that if you don't know it, don't pretend that you do but let the kids know that, "well, look, I don't know about that, but I've got a phone number I can phone" (Ian, foster carer)*	Institutional	Facilitated SHR discussions by providing caregivers with resources they could access and use with LAC	Reduced role overload
F5	Training highlighted that SHR discussions were routinely happening in care<br>*"we were playing Connect 4, and one of the girls said 'how do you get pregnant' and we said*	Personal	No perceived effect reported by	Contributed to role overload in

For peer review only - http://bmjopen.bmj.com/site/about/guidelines.xhtml

'well, you need to have sex'. 'Aye, I know that… and I know that he cums, but how does that then work?' So we dismantled the Connect 4, and we said 'well it's no square, but you'll have to imagine this is a womb, and these are the fallopian tubes', and we used the wee circles as the sperms and the eggs, and we used that to explain it…. Once we were finished I turned to [another caregiver] and said 'did I get that right?'" (Claire, residential carer).<br><br>*"I remember, certainly, a few years ago having a discussion with an 18 year old girl who wasn't sure what she looked like, errm, err, down below. What her vulva looked like. And about sex, sexual intercourse. She didn't know whether she would be able to partake in that and I do, really remember, just frankly saying to her, why don't you just get a mirror and have a look, you know, oh I can't be doing that, but why can't you be doing that, it's the easiest way to kind of look and have a see, to explore your own bodies and you'll know what's likeable, what's not likeable, what you're happy with people to touch and what you're not happy for people to touch" (Tricia, residential care)*		caregivers	some cases by creating anxiety that SHR information being provided was correct
**Pastoral support**			
G1 Support of management<br>*"I found that work really difficult, because I had never had to deal with… trying to manage a child – cos he was a child at the time — who is not only, you know, being abused, but is an abuser… I felt really, you know, unsure of how best to manage that. One of the best things with managing that was that my manager agreed to support me, and we did the work together" (Agnes, social worker)*	Institutional	Facilitated SHR discussions by providing caregivers with additional supports	Reduced role overload
G2 Peer supervision<br>*"We quite often in this team have group supervision… where I might not have had the experience of working with a young person in that situation for a couple of months, someone else probably has or will have without doubt, so it's about other people sharing their experiences and information and sometimes that's the best way to learn because you are speaking about real experiences and examples (Agnes, social worker).*<br><br>*"We deal with it pretty well, but I think with this wee core group of carers that we've got there's always an opportunity for learning… 'I've tried to get this boy to do his bloody homework and he just will not do it' and somebody will say 'try this' and you find that it works. That's where our support is… from other carers in our group. We bounce off each other" (Ian, foster carer)*	Institutional	Facilitated SHR discussions by providing caregivers with additional supports and continued informal learning	Reduced role overload

For peer review only - http://bmjopen.bmj.com/site/about/guidelines.xhtml

References

1. Geiger, J.M. and L.A. Schelbe, *Stopping the cycle of child abuse and neglect: A call to action to focus on pregnant and parenting youth in and aging out of the foster care system.* Journal of Public Child Welfare, 2014. **8**(1): p. 25-50.
2. *Children Act*. 1989, HMSO: London.
3. The Scottish Government, *Children's Social Work Statistics 2015-16*. 2017, The Scottish Government: Edinburgh.
4. Information Analysis Directorate, *Children's Social Care Statistics for NI 2016/17*. 2017, Department of Health: Stormont.
5. Welsh Government, *Children looked after by local authorities, 2016-17 (Experimental statistics)*. 2017.
6. Department of Education, *Children looked after in England including adoption: 2016 to 2017*. 2017, The Stationery Office: London.
7. The Scottish Government, *Children's Social Work Statistics Scotland, 2013-14*. 2015: Edinburgh.
8. Pecora, P.J., et al., *Mental health of current and former recipients of foster care: A review of recent studies in the USA.* Child & Family Social Work, 2009. **14**(2): p. 132-146.
9. Vinnerljung, B. and M. Sallnäs, *Into adulthood: a follow-up study of 718 young people who were placed in out-of-home care during their teens.* Child & Family Social Work, 2008. **13**(2): p. 144-155.
10. Hjern, A., F. Lindblad, and B. Vinnerljung, *Suicide, psychiatric illness, and social maladjustment in intercountry adoptees in Sweden: a cohort study.* The Lancet, 2002. **360**(9331): p. 443-448.
11. Vinnerljung, B., M. Öman, and T. Gunnarson, *Educational attainments of former child welfare clients–a Swedish national cohort study.* International Journal of Social Welfare, 2005. **14**(4): p. 265-276.
12. Dube, S.R., et al., *The impact of adverse childhood experiences on health problems: evidence from four birth cohorts dating back to 1900.* Preventive medicine, 2003. **37**(3): p. 268-277.
13. Bonell, C., et al., *The effect of dislike of school on risk of teenage pregnancy: testing of hypotheses using longitudinal data from a randomised trial of sex education.* Journal of Epidemiology and Community Health, 2005. **59**(3): p. 223-230.
14. Ellis, B.J., et al., *Does father absence place daughters at special risk for early sexual activity and teenage pregnancy?* Child Development, 2003. **74**(3): p. 801-821.
15. Ramrakha, S., et al., *Psychiatric disorders and risky sexual behaviour in young adulthood: cross sectional study in birth cohort.* BMJ, 2000. **321**(7256): p. 263-266.
16. Otto-Salaj, L.L., et al., *Psychiatric functioning and substance use: Factors associated with HIV risk among incarcerated adolescents.* Child Psychiatry and Human Development, 2002. **33**(2): p. 91-106.
17. Carpenter, S.C., et al., *The association of foster care or kinship care with adolescent sexual behavior and first pregnancy.* Pediatrics, 2001. **108**(3): p. e46-e46.
18. Ahrens, K.R., et al., *Laboratory-diagnosed sexually transmitted infections in former foster youth compared with peers.* Pediatrics, 2010. **126**(1): p. e97-e103.
19. Dworsky, A. and M.E. Courtney, *The risk of teenage pregnancy among transitioning foster youth: Implications for extending state care beyond age 18.* Children and Youth Services Review, 2010. **32**(10): p. 1351-1356.
20. Vinnerljung, B., E. Franzén, and M. Danielsson, *Teenage parenthood among child welfare clients: A Swedish national cohort study of prevalence and odds.* Journal of Adolescence, 2007. **30**(1): p. 97-116.
21. Henderson, M., et al., *Heterosexual risk behaviour among young teenagers in Scotland.* Journal of Adolescence, 2002. **25**(5): p. 483-494.

For peer review only - http://bmjopen.bmj.com/site/about/guidelines.xhtml

22. Ahrens, K.R., et al., *Association between childhood sexual abuse and transactional sex in youth aging out of foster care.* Child Abuse & Neglect, 2012. **36**(1): p. 75-80.
23. Ahrens, K.R., et al., *Psychosocial Pathways to Sexually Transmitted Infection Risk Among Youth Transitioning Out of Foster Care: Evidence From a Longitudinal Cohort Study.* Journal of Adolescent Health, 2013. **53**(4): p. 478-485.
24. Dale, H., et al., *The perceived sexual health needs of looked after young people: findings from a qualitative study led through a partnership between public health and health psychology.* Journal of Public Health, 2011. **33**(1): p. 86-92.
25. Knight, A., E. Chase, and P. Aggleton, *'Someone of Your Own to Love': Experiences of Being Looked After as Influences on Teenage Pregnancy.* Children & Society, 2006. **20**(5): p. 391-403.
26. McFreely, S., *Teenage Pregnancy in Looked After Young People.* Practice Nurse

2003. **29**(37-42).
27. Love, L.T., et al., *Fostering hope: preventing teen pregnancy among youth in foster care.* Gender and Development, 2005. **13**(3): p. 34-41.
28. Constantine, W.L., P. Jerman, and N.A. Constantine, *Sex education and reproductive health needs of foster and transitioning youth in three California counties.* Public Health Institute. March, 2009. **2**.
29. Lipscombe, J., E. Farmer, and S. Moyers, *Parenting fostered adolescents: skills and strategies.* Child & Family Social Work, 2003. **8**(4): p. 243-255.
30. Bruce, J.S., *Sexual and reproductive health policies for foster youth in California: A qualitative study of child welfare professionals' experiences and perceptions of policies.* Children and Youth Services Review, 2016. **61**: p. 184-200.
31. Fullerton, D., et al., *Sexual Health and Sexuality Education Needs Assessment of Young People in Care in Ireland (SENYPIC): A Survey of Service-provider Perspectives. Report No. 1.* 2016, HSE Crisis Pregnancy Programme and the Child & Family Agency: Dublin.
32. Boonstra, H.D., *Teen pregnancy among young women in foster care: A primer.* Guttmacher Policy Review, 2011. **14**(2).
33. Winter, V.R., R.A. Brandon-Friedman, and G.E. Ely, *Sexual health behaviors and outcomes among current and former foster youth: A review of the literature.* Children and Youth Services Review, 2016. **64**: p. 1-14.
34. DiClemente, R.J., et al., *Parent-adolescent communication and sexual risk behaviors among African American adolescent females.* The Journal of pediatrics, 2001. **139**(3): p. 407-412.
35. Stone, N. and R. Ingham, *Factors affecting British teenagers' contraceptive use at first intercourse: The importance of partner communication.* Perspectives on Sexual and Reproductive Health, 2002: p. 191-197.
36. Hutchinson, M.K., et al., *The role of mother–daughter sexual risk communication in reducing sexual risk behaviors among urban adolescent females: a prospective study.* Journal of adolescent health, 2003. **33**(2): p. 98-107.
37. DiIorio, C., E. Pluhar, and L. Belcher, *Parent-child communication about sexuality: A review of the literature from 1980–2002.* Journal of HIV/AIDS Prevention & Education for Adolescents & Children, 2003. **5**(3-4): p. 7-32.
38. Healthcare Policy and Strategy Directorate: Child and Maternal Health Division, *CEL-16: Implementation of action 15 of the looked after children and young people: we can and must do better report*. 2009, The Scottish Government,: Edinburgh.
39. The Scottish Government, *Better Health, Better Care: Action Plan*. 2007, HMSO: Edinburgh.
40. Stryker, S., *Symbolic interactionism: A social structural version*. 1980: Blackburn Press Caldwell,, NJ.
41. Stryker, S. and R.T. Serpe, *Identity salience and psychological centrality: Equivalent, overlapping, or complementary concepts?* Social Psychology Quarterly, 1994: p. 16-35.

For peer review only - http://bmjopen.bmj.com/site/about/guidelines.xhtml

42. Stryker, S. and A. Statham, *Symbolic interaction and role theory*, in *The handbook of social psychology* G. Lindzey and E. Aronson, Editors. 1985, Random House: New York. p. 311-378.
43. Brookes, K., et al., *Role theory: A framework to investigate the community nurse role in contemporary health care systems.* Contemporary nurse, 2007. **25**(1-2): p. 146-155.
44. McKenna, H., S. Keeney, and M. Bradley, *Generic and specialist nursing roles in the community: an investigation of professional and lay views.* Health & social care in the community, 2003. **11**(6): p. 537-545.
45. Brown, B., P. Crawford, and J. Darongkamas, *Blurred roles and permeable boundaries: the experience of multidisciplinary working in community mental health.* Health & Social Care in the Community, 2000. **8**(6): p. 425-435.
46. Major, D.A., *Utilizing role theory to help employed parents cope with children's chronic illness.* Health education research, 2003. **18**(1): p. 45-57.
47. Gardner, J.S., et al., *Pharmacist prescribing of hormonal contraceptives: results of the Direct Access study.* Journal of the American Pharmacists Association: JAPhA, 2007. **48**(2): p. 212-21; 5 p following 221.
48. Austin, W., et al. *Moral distress in healthcare practice: The situation of nurses*. in *HEC Forum*. 2005. Springer.
49. Pomaki, G., A. Supeli, and C. Verhoeven, *Role conflict and health behaviors: Moderating effects on psychological distress and somatic complaints.* Psychology and Health, 2007. **22**(3): p. 317-335.
50. McLean, J., *Men as Minority Men Employed in Statutory Social Care Work.* Journal of Social Work, 2003. **3**(1): p. 45-68.
51. BBC News. *Child protection 'overstretched'*. 2009 13/07/2009 23/01/2019]; Available from: http://news.bbc.co.uk/1/hi/scotland/8148361.stm.
52. O'Brien, B., et al., *Standards for reporting qualitative research: a synthesis of recommendations.* Acad Med 2014. **89**(9): p. 1245-1251.
53. Gregory, A., et al., *School climate and implementation of a preventive intervention.* American journal of community psychology, 2007. **40**(3-4): p. 250-260.
54. The Scottish Government, *These are our bairns: a guide for community planning partnerships on being a good corporate parent*. 2008, Scottish Government Edinburgh.
55. East, L.J. and T.R. Orchard, *Somebody else's job: Experiences of sex education among health professionals, parents and adolescents with physical disabilities in Southwestern Ontario.* Sexuality and Disability, 2014. **32**(3): p. 335-350.
56. Sugerman, S., et al., *Family planning clinic patients: their usual health care providers, insurance status, and implications for managed care.* Journal of Adolescent Health, 2000. **27**(1): p. 25-33.
57. The Scottish Government, *Decision Making Guidance for Foster Carers*. 2015: Edinburgh.
58. Ruane, J. and M. Hayter, *Nurses' attitudes towards sexual relationships between patients in high security psychiatric hospitals in England: An exploratory qualitative study.* International Journal of Nursing Studies, 2008. **45**(12): p. 1731-1741.
59. Higgins, A., P. Barker, and C.M. Begley, *'Veiling sexualities': a grounded theory of mental health nurses responses to issues of sexuality.* Journal of Advanced Nursing, 2008. **62**(3): p. 307-317.
60. Pound, P., et al., *What is best practice in sex and relationship education? A synthesis of evidence, including stakeholders' views.* BMJ Open, 2017. **7**(5).
61. Farmer, E., J. Lipscombe, and S. Moyers, *Foster Carer Strain and its Impact on Parenting and Placement Outcomes for Adolescents.* British Journal of Social Work, 2005. **35**(2): p. 237-253.
62. Dane, B., *Child Welfare Workers.* Journal of Social Work Education, 2000. **36**(1): p. 27-38.
63. Rhodes, K.W., et al., *Foster family resources, psychosocial functioning, and retention.* Social work research, 2003. **27**(3): p. 135-150.

For peer review only - http://bmjopen.bmj.com/site/about/guidelines.xhtml

64. Unrau, Y.A., J.R. Seita, and K.S. Putney, *Former foster youth remember multiple placement moves: A journey of loss and hope.* Children and Youth Services Review, 2008. **30**(11): p. 1256-1266.
65. Knight, A., E. Chase, and P. Aggleton, *Teenage Pregnancy among Young People in and Leaving Care: Messages and Implications for Foster Care.* Adoption & Fostering, 2006. **30**(1): p. 58-69.
66. James, S., et al., *Sexual risk behaviors among youth in the child welfare system.* Children and Youth Services Review, 2009. **31**(9): p. 990-1000.
67. Dvir, Y., et al., *Childhood maltreatment, emotional dysregulation, and psychiatric comorbidities.* Harvard review of psychiatry, 2014. **22**(3): p. 149.
68. Hoagwood, K.E., et al., *Family support in children's mental health: A review and synthesis.* Clinical child and family psychology review, 2010. **13**(1): p. 1-45.
69. Quinn, C., B. Happell, and G. Browne, *Talking or avoiding? Mental health nurses' views about discussing sexual health with consumers.* International journal of mental health nursing, 2011. **20**(1): p. 21-28.
70. Buston, K., et al., *Implementation of a teacher-delivered sex education programme: obstacles and facilitating factors.* Health Education Research, 2002. **17**(1): p. 59-72.
71. Faulder, G.S., et al., *Teaching sex education improves medical students' confidence in dealing with sexual health issues.* Contraception, 2004. **70**(2): p. 135-139.
72. Jaccard, J., P.J. Dittus, and V.V. Gordon, *Parent-Teen Communication about Premarital Sex Factors Associated With the Extent of Communication.* Journal of Adolescent Research, 2000. **15**(2): p. 187-208.
73. Oakley, A., *Interviewing women: A contradiction in terms*, in *Doing feminist research*. 2013, Routledge. p. 52-83.
74. Reinharz, S., *Who am I? The need for a variety of selves in the field.* Reflexivity and voice, 1997: p. 3-20.
75. Elliott, L., C. Kennedy, and R. Raeside, *Professional role identity in shaping community nurses' reactions to nursing policy.* Journal of nursing management, 2015. **23**(4): p. 459-467.

For peer review only

For peer review only - http://bmjopen.bmj.com/site/about/guidelines.xhtml

Supplementary File 1: Interview Schedule

Introductions

Can you tell me a little bit about your experience working with/caring for looked after young people?

Explore: Type of contact with young people

Length of time working with them

Current Role

Reasons for working with the group

Views of sexual health issues affecting young people in care

Thinking about sexual health, can you tell me what you think are the most important issue affecting young people in care?

Explore: Pubertal changes, gender difference and menarche

Emotions and self esteem

Boyfriends and girlfriends

Choosing when to have sex and delaying sex

Contraception

STIs

Teenage pregnant/parenthood

Circumstances surrounding sex (e.g. risky, drugs/alcohol, force, consent)

Regret

Sexual health education

Service use

For peer review only

What/who do you think influences the sexual health of young people in care?

Explore: Type of care placement

Stability of placement

Security

Influence of peers/partners/parents/carers/corporate family

Media

Other

Sexual health education

Where do you think that looked after young people learn about sexual health and relationships?

Explore: School

Family

Friends/peers

Media

Social workers

Carers

Other

Thinking about the looked after young people and care leavers you know of look after, do you know what they know about sexual health and relationships?

Explore: What do they know?

What don't they know?

Where have they learned that information from?

What do you think about the quality of the information they received?

Have you spoken to them about sexual health and relationships?

- Why/Why not?

What have you spoken to them about?

For peer review only

Do you think there are barriers to young people in care accessing sexual health and relationships education?

Explore: What are those barriers?

What could be done to overcome the barriers?

Whose responsibility should that be?

Do you think that it is important for LAC to learn about sexual health and relationships?

Explore: Why/why not?

What do you think are the most important things for them to learn about? Why?

What would you change about the way young people in care learn about sexual health and relationships? Why do you think that?

<u>Help, support and guidance around sexual health and relationships</u>

What support should young people in care need in relation to their sexual health and relationships?

Explore: Pubertal changes and menarche

Boyfriends/girlfriends

Relationships

Choosing to have sex and sexual delay

Getting and using contraception

Using sexual health services

Pregnancy

Abortion

Other topics/advice/kinds of support not mentioned

Have you ever delivered support to a young person in care/care leaver in relation to their sexual health? Can you tell me about that in more detail?

How did delivering that support make you feel?

For peer review only

Did you feel that you had received sufficient training to be able to talk to a young person about their sexual health?

Explore: What training/support have you received?

What training/support would you have needed?

Who should have given you that support?

What effect do you think the training had/would have had upon your ability to support the young person you were speaking to?

What effect do you think you advice had upon the young person's…

Explore: Self confidence and self esteem

Knowledge

Relationships with boyfriends/girlfriends

Sexual relationships

Their relationship with you

Sexual health service use

What do you think are the barriers to young people in care accessing sexual health services?

If a young person came to you asking for information about sexual health services, could you give the that information? Where would you direct them to? Why? Would you accompany them there if they wanted you to?

What do you think a sexual health service for looked after young people should be like?

Explore: Staff

Accessibility

Opening hours/appointments

Location/entrance

Confidentiality

Other

For peer review only

Improving outcomes

What could be done to promote positive sexual health outcomes for looked after young people?

Why do you think that?

For peer review only

SRQR checklist for manuscript: Providing sex and relationships education for looked after children: a qualitative exploration of how personal and institutional factors promote or limit the experience of role ambiguity conflict and overload among caregivers

		Reporting Item	Page Number
Problem formulation	#1	Concise description of the nature and topic of the study identifying the study as qualitative or indicating the approach (e.g. ethnography, grounded theory) or data collection methods (e.g. interview, focus group) is recommended	2-3, 6
	#2	Summary of the key elements of the study using the abstract format of the intended publication; typically includes background, purpose, methods, results and conclusions	2-3
	#3	Description and significance of the problem / phenomenon studied: review of relevant theory and empirical work; problem statement	4-6
Purpose or research question	#4	Purpose of the study and specific objectives or questions	6
Qualitative approach and research paradigm	#5	Qualitative approach (e.g. ethnography, grounded theory, case study, phenomenology, narrative research) and guiding theory if appropriate; identifying the research paradigm (e.g. postpositivist, constructivist / interpretivist) is also recommended; rationale. The rationale should briefly discuss the justification for choosing that theory, approach, method or technique rather than other options available; the assumptions and limitations implicit in those choices and how those choices influence study conclusions and transferability. As appropriate the rationale for several items might be discussed together.	8-9
Researcher characteristics and reflexivity	#6	Researchers' characteristics that may influence the research, including personal attributes, qualifications / experience, relationship with participants, assumptions and / or presuppositions; potential or actual interaction between researchers'	26-27

For peer review only

		characteristics and the research questions, approach, methods, results and / or transferability	
Context	#7	Setting / site and salient contextual factors; rationale	7
		Reporting Item	Page Number
Sampling strategy	#8	How and why research participants, documents, or events were selected; criteria for deciding when no further sampling was necessary (e.g. sampling saturation); rationale	7
Ethical issues pertaining to human subjects	#9	Documentation of approval by an appropriate ethics review board and participant consent, or explanation for lack thereof; other confidentiality and data security issues	9, 31
Data collection methods	#10	Types of data collected; details of data collection procedures including (as appropriate) start and stop dates of data collection and analysis, iterative process, triangulation of sources / methods, and modification of procedures in response to evolving study findings; rationale	8-9
Data collection instruments and technologies	#11	Description of instruments (e.g. interview guides, questionnaires) and devices (e.g. audio recorders) used for data collection; if / how the instruments(s) changed over the course of the study	8, Supplementary File 1
Units of study	#12	Number and relevant characteristics of participants, documents, or events included in the study; level of participation (could be reported in results)	7-8
Data processing	#13	Methods for processing data prior to and during analysis, including transcription, data entry, data management and security, verification of data integrity, data coding, and anonymisation / deidentification of excerpts	8-9
Data analysis	#14	Process by which inferences, themes, etc. were identified and developed, including the researchers involved in data analysis; usually references a specific paradigm or approach; rationale	8-9
Techniques to enhance trustworthiness	#15	Techniques to enhance trustworthiness and credibility of data analysis (e.g. member checking, audit trail, triangulation); rationale	8-9

For peer review only

Syntheses and interpretation	#16	Main findings (e.g. interpretations, inferences, and themes); might include development of a theory or model, or integration with prior research or theory	10-21
Links to empirical data	#17	Evidence (e.g. quotes, field notes, text excerpts, photographs) to substantiate analytic findings	10-21, Table 1
		Reporting Item	Page Number
Intergration with prior work, implications, transferability and contribution(s) to the field	#18	Short summary of main findings; explanation of how findings and conclusions connect to, support, elaborate on, or challenge conclusions of earlier scholarship; discussion of scope of application / generalizability; identification of unique contributions(s) to scholarship in a discipline or field	22-25
Limitations	#19	Trustworthiness and limitations of findings	26-28
Conflicts of interest	#20	Potential sources of influence of perceived influence on study conduct and conclusions; how these were managed	30
Funding	#21	Sources of funding and other support; role of funders in data collection, interpretation and reporting	30

For peer review only
